# Dynamic structural plasticity facilitates the developmental assembly of cochlear inner hair cell ribbon synapses

**DOI:** 10.3389/fcell.2026.1866692

**Published:** 2026-09-04

**Authors:** Roos A. Voorn, Michael Sternbach, Jérôme Bourien, Noboru H. Komiyama, Vladan Rankovič, Fred Wolf, Seth G. N. Grant, Christian Vogl

**Affiliations:** 1 Auditory Neuroscience Group, Institute of Physiology, Medical University Innsbruck, Innsbruck, Austria; 2 Presynaptogenesis and Intracellular Transport in Hair Cells Junior Research Group, Institute for Auditory Neuroscience and InnerEarLab, University Medical Center Goettingen, Goettingen, Germany; 3 Collaborative Research Center 889 “Cellular Mechanisms of Sensory Processing”, Goettingen, Germany; 4 Göttingen Graduate Center for Neurosciences, Biophysics and Molecular Biosciences, Goettingen, Germany; 5 Max Planck Institute for Dynamics and Self-Organization, Goettingen, Germany; 6 Göttingen Campus Institute for Dynamics of Biological Networks, University of Göttingen, Goettingen, Germany; 7 INM, University Montpellier, INSERM, Montpellier, France; 8 Genes to Cognition Program, Centre for Clinical Brain Sciences, University of Edinburgh, Edinburgh, United Kingdom; 9 Simons Initiative for the Developing Brain, Centre for Discovery Brain Sciences, University of Edinburgh, Edinburgh, United Kingdom; 10 The Patrick Wild Centre for Research into Autism, Fragile X Syndrome and Intellectual Disabilities, Centre for Discovery Brain Sciences, University of Edinburgh, Edinburgh, United Kingdom; 11 Muir Maxwell Epilepsy Centre, University of Edinburgh, Edinburgh, United Kingdom; 12 Institute for Auditory Neuroscience and InnerEarLab, University Medical Center Goettingen, Goettingen, Germany; 13 Bernstein Center for Computational Neuroscience, Goettingen, Germany

**Keywords:** active zone maturation, auditory hair cells, ribbon synapse, sensory systems, synaptogenesis

## Abstract

Sound detection occurs in the cochlea, where sensory inner hair cells (IHC) accurately convert auditory stimuli into neurochemical signals. Presynaptically, IHCs harbor synaptic ribbons, specialized scaffolds that facilitate ultrafast and indefatigable exocytosis. During synapse assembly and subsequent maturation, IHC ribbons increase in volume and synaptic vesicle tethering capacity. This development is thought to result from progressive precursor aggregation. However, the underlying mechanisms of ribbon synapse formation have remained elusive thus far. In this study, we established a novel triple-color live-cell imaging approach to monitor IHC presynaptogenesis *in situ*. We found that ribbon precursors are highly dynamic and undergo bidirectional plasticity. The presynaptic active zone (AZ) forms a focal point for dramatic structural remodeling of precursors, which the AZ recruits, confines and redistributes. Furthermore, silencing spontaneous synaptic activity decreased precursor mobility and plasticity at the AZ. This suggests a fundamental role for activity-dependent Ca^2+^ influx in regulating the dynamic assembly of auditory ribbon synapses.

## 1 Introduction

Ultrafast synaptic sound encoding translates physical sound waves into neural code, thus enabling speech perception and spatial orientation in a three-dimensional environment. In mammals, this challenging task is performed by “ribbon-type” synapses between cochlear inner hair cells (IHC) and postsynaptic spiral ganglion neurons (SGN) that ensure temporally-precise and indefatigable signal transduction. Ribbons play a fundamental role in the recruitment, priming, and replenishment of synaptic vesicles (SVs), and may further serve as a diffusion barrier for presynaptic Ca^2+^ at the active zone (AZ) (Zenisek et al., 2004; Khimich et al., 2005; LoGiudice et al., 2008; Graydon et al., 2011; Snellman et al., 2011; Maxeiner et al., 2016; Becker et al., 2018; Jean et al., 2018). In fact, ribbons are thought to fulfill a multifaceted scaffolding role at the cytomatrix of the AZ (CAZ), where they tether glutamate-filled SVs and cluster presynaptic Ca_v_1.3 channels—thus facilitating ultrafast and highly efficient excitation-secretion coupling (Frank et al., 2010; Sheets et al., 2011).

While various studies have investigated the unique structure and physiology of mature IHC ribbon synapses, knowledge on the early development remains limited to date. Yet, previous findings indicate a dynamic process in which small cytoplasmic ribbon precursors gradually accumulate at the assembling AZ during late embryogenesis and gradually fuse to form mostly single, membrane-anchored ribbons at hearing onset (Sobkowicz et al., 1986; Wong et al., 2014; Michanski et al., 2019). This structural transition period is currently thought to involve molecular motor driven re-distribution of ribbon material that largely concludes around P12 (Michanski et al., 2019; Voorn et al., 2024). However, the exact underlying molecular mechanisms as well as the regulatory pathways remain to be determined. Notably, it is well established that developing organs of Corti intrinsically generate spontaneous activity (SA) waves, which trigger sensory-independent IHC exocytosis to initiate auditory pathway activation (Beutner and Moser, 2001; Tritsch et al., 2010). This pre-sensory SA has previously been proven essential for the diversification and survival of SGNs, as well as the maturation of ribbon synapse function through the tightening of Ca^2+^ influx to exocytosis coupling (Johnson et al., 2013; Wong et al., 2014; Zhang-Hooks et al., 2016; Shrestha et al., 2018; Sun et al., 2018). In addition, work on developing zebrafish lateral line neuromast hair cells indicated the importance of adequate presynaptic Ca^2+^ influx for ribbon size regulation during maturation (Sheets et al., 2011). In line with these earlier works, we recently demonstrated that homeostatic scaling of developing IHC ribbon synapses can be triggered by imposed deviations from the native SA pattern (Halim et al., 2026). Considering the activity-induced adaptations in ribbon structure that have been reported in other sensory systems and across a range of species (Hermes et al., 1992; Adly et al., 1999; Spiwoks-Becker et al., 2004; Spiwoks-Becker et al., 2008; Dembla et al., 2020), it is therefore highly likely that the pre-sensory SA in the organ of Corti plays a fundamental role in the maturational refinement of the presynaptic architecture of IHC ribbon synapses.

To now experimentally address these issues, we set out to investigate IHC ribbon synapse developmental plasticity in real time during the critical early postnatal period. For this purpose, we devised a multi-color live-cell imaging paradigm to monitor the structural dynamics of IHC pre- and postsynaptic components *in situ* using short-term organotypically-cultured cochlear explants as a model system and combined this with Ca^2+^ imaging, immunohistochemistry and optical nanoscopy to comprehensively characterize the intrinsic structural plasticity of IHC synapses during postnatal development. We recently found that auditory ribbon synapse assembly is a highly dynamic process, in which ribbon precursors are subject to bidirectional plasticity events—i.e., precursor fusion and fission (Voorn et al., 2024). Here, we show that the presynaptic AZ serves as a plasticity hub, where ribbon precursors can be recruited and stabilized, yet also locally re-distributed or even exchanged between distant AZs in a strikingly targeted manner. Moreover, the observed structural plasticity appears to depend on synapse location within the IHC basolateral compartment and is further regulated by SA-driven presynaptic Ca^2+^ influx.

## 2 Materials and methods

### 2.1 Animals

All animal experiments were conducted in accordance with national as well as regional guidelines of Lower Saxony, Germany and were approval by the Lower Saxony animal welfare officer. For our experiments, PSD95-HaloTag (Masch et al., 2018; Bulovaite et al., 2022) mice were crossbred with Ai32-Vglut3-Cre Knock-In mice (Ai32-VC-KI-PSD (Madisen et al., 2010; Vogl et al., 2015; Chakrabarti et al., 2022; Voorn et al., 2024); Ai32: JAX#024109), or Ai14-Neurogenin1-Cre/ERT2 mice (Ai14-Neurog1-PSD (Koundakjian et al., 2007; Madisen et al., 2010); Ai14: JAX#007914; Neurogenin1-Cre: JAX#008529). Furthermore, C57Bl6/J wild-type mice (WT), and SNAP25-2A-GCaMP6s-D mice (SNAP25-GCaMP6 (Madisen et al., 2015); SNAP25: JAX#025111) were used (Table 1). Mice of either sex between the ages of postnatal day (P)5 and 7 were sacrificed by decapitation for preparation of organotypic cultures of the organ of Corti.

**TABLE 1:** Key resources table.

Reagent or resource	Source	Identifier
Antibodies		
Mouse IgG2a α-β3-Tubulin (Tuj-1)	BioLegend	#801202
Chicken α-Bassoon	Synaptic Systems	#141016
Mouse IgG2a α-Bassoon	Abcam	#ab82958
Mouse IgG1 α-CtBP2	Becton Dickinson	#612044
Mouse IgG1 α-Myosin VIIa	DSHB	#MYO7A 138-1
Rabbit α-Piccolino (αAczp18p19)	a kind gift of Prof. Manfred Kilimann (Limbach et al., 2011)	N/A
Mouse IgG1 α-PSD95, clone 7E3-1B8	Sigma	#MAB1598
Rabbit α-RIBEYE-A	Synaptic Systems	#192103
Goat α-Chicken AlexaFluor647	Abcam	#ab150171
Goat α-Mouse Star580	Abberior	#ST580-1001-500UG
Goat α-Mouse IgG1 AlexaFluor488	Life Technologies	#A21121
Goat α-Mouse IgG1 AlexaFluor594	Life Technologies	#A21125
Camelid α-Mouse IgG1 Star635P FluoTag®-X2	NanoTag	#N2002-Ab635P
Camelid α-Mouse IgG2 Atto565 FluoTag®-X2	NanoTag	#N2702-At565
Camelid α-Mouse IgG2 Star635P FluoTag®-X2	NanoTag	#N2702-Ab635P
Camelid α-Rabbit Star580 FluoTag®-X4	NanoTag	#N2404
Goat α-Rabbit Star635P	Abberior	#ST635P-1002-500UG
Camelid α-GFP Atto488 FluoTag®-X4	NanoTag	#N0304-At488
Camelid α-PSD95 Atto488 FluoTag®-X2	NanoTag	#N3702-At488
Camelid α-PSD95 AlexaFluor647 FluoTag®-X2	NanoTag	#N3702-AF647
Camelid α-RFP Atto565 FluoTag®-X4	NanoTag	#N0404-At565
Virus strains		
PHP.B_CMV-HBA-RIBEYE-GFP_WRPE_bGH	Addgene; Voorn et al. (Voorn et al., 2024)	pUCmini-iCAP-PHP.eB plasmid: RRID:Addgene_103005
PHP.eB_CMV-HBA_RIBEYE-tdTomato_WPRE_ bGH	Addgene; Voorn et al. (Voorn et al., 2024)	pUCmini-iCAP-PHP.eB plasmid: RRID:Addgene_103005
PHP.B_HBA_Katushka-RIBEYE-BP_WPRE_bGHpA	Addgene; This paper	pUCmini-iCAP-PHP.eB plasmid: RRID:Addgene_103005
Chemicals, peptides, and recombinant proteins		
Ampicillin	Sigma	A0166
B27	Life Technologies	17504044
CellTak	Corning	#354240
Dimethyl sulfoxide Hybri-Max^TM^	Sigma	D2650
Fungizone	Life Technologies	15290-026
GlutaMAX^TM^-1 (100X)	Gibco	35050-061
HBSS (1X)	Gibco	14025-092
HEPES	Gibco	15630-049
Isradipine	Tocris	#2004
Immersol immersion oil Ne 1.33	Zeiss	W2010
Janelia Fluor® 549 HaloTag® Ligand	Promega	#GA1110
Janelia Fluor® 646 HaloTag® Ligand	Promega	#GA1120
Neurobasal-A medium (1X)	Gibco	12349-015
ProLong™ Glass Antifade Mountant	Life Technologies	P36984
Penicillin G	Sigma	P3032
Deposited data		
Raw and analyzed data	This paper	Raw data deposited on Mendeley Data, V1, doi: 10.17632/cktzhykhxf.1
Experimental models: organisms/strains		
Mouse: Ai32-Vglut3-Cre Knock-In-PSD95-HaloTag	Ai32-VC-KI (Madisen et al., 2010; Vogl et al., 2015; Chakrabarti et al., 2022; Voorn et al., 2024), PSD95-Halotag (Masch et al., 2018; Bulovaite et al., 2022)	Ai32: JAX#024109
Mouse: Ai14-Neurogenin1-Cre/ERT2 –PSD95-HaloTag	Ai14-Neurog1-PSD (Koundakjian et al., 2007; Madisen et al., 2010)	Ai14: JAX#007914
Mouse: B6.Cg-*Snap25* ^*tm3.1Hze*^/J (Snap25-2A-GCaMP6s-D)	SNAP25-GCaMP6 (Madisen et al., 2015)	SNAP25: JAX#025111
Mouse: C57Bl6/J	The Jackson Laboratory	JAX ID: #000664
Software and algorithms		
ImageJ/FIJI	Schneider et al. (2012)	https://imagej.nih.gov/ij
Imaris (x64 9.6.1)	Oxford Instruments	imaris.oxinst.com
Custom MATLAB script for MSD calculations	Voorn et al. (2024)	​
Prism8	GraphPad, San Diego, CA	​
BleachCorrection	CorrectBleach (Miura, 2020)	​

### 2.2 Organotypic culture preparation of the mouse organ of Corti

**FIGURE 1:**
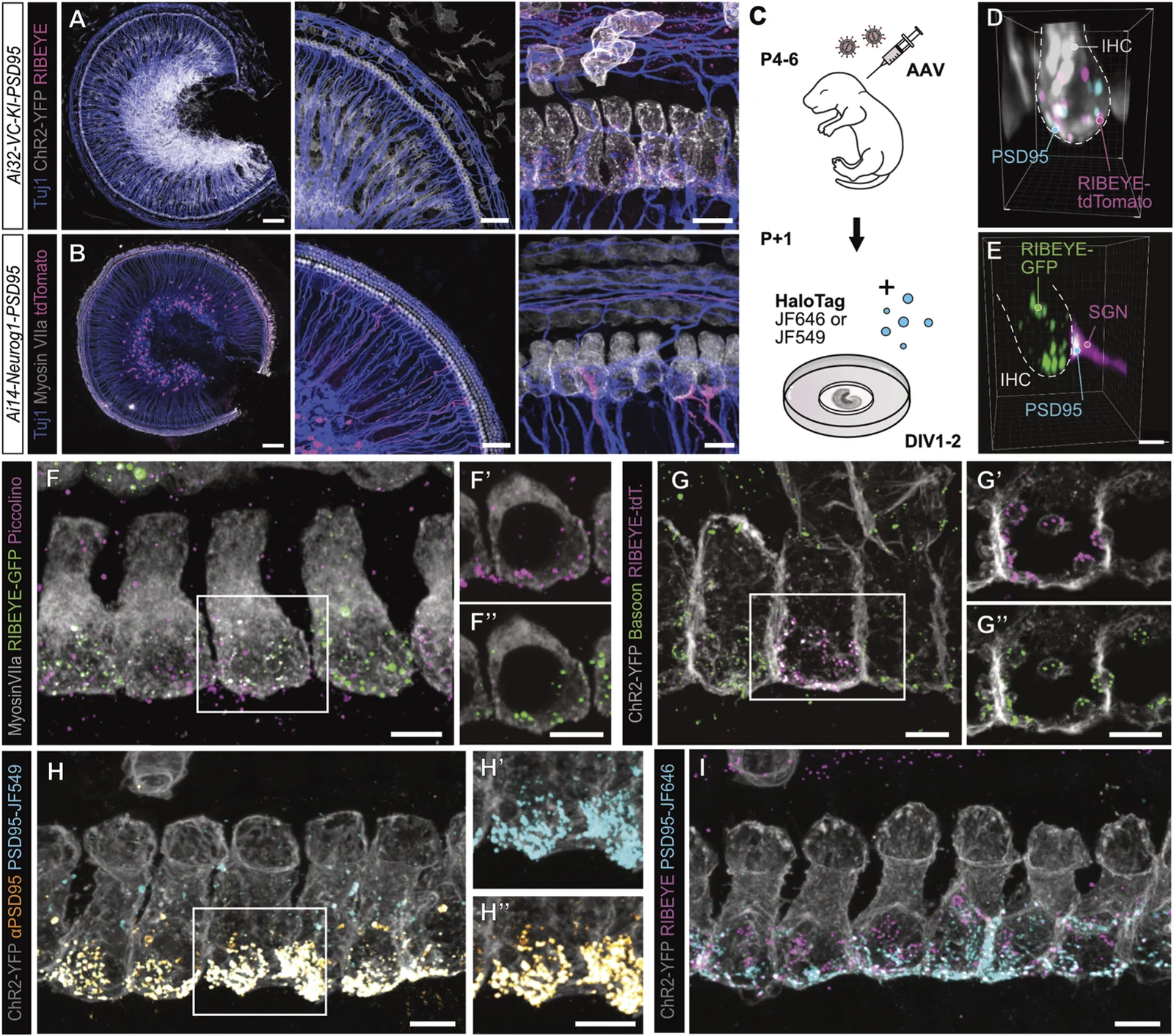
Experimental setup for the tracing of IHC ribbon precursor dynamics in neonatal mice. **(A)** Tissue overview of an organotypic culture of the organ of Corti of an Ai32-VC-KI-PSD mouse with endogenous YFP fluorescence of the IHC plasma membrane (exemplary images of cultures P6DIV2, P6DIV2, P5DIV1). **(B)** Tissue overview of an Ai14-Neurog1-PSD95 mouse with endogenous sparse labeling of SGNs with tdTomato (exemplary images of cultures P5DIV1, P5DIV3, P7DIV1). For long-term time-lapse imaging of SGN innervation in the Ai14-Neurog1-PSD line, see Supplementary Video S1. **(C)** Experimental setup, making use of the mouse lines in **(A)** and **(B)**. Viral injections of a RIBEYE-XFP AAV (either -GFP or -tdTomato) are performed in neonatal mice, upon which the organ of Corti is extracted and organotypically cultured 1 day post injection. *In vitro* application of a HaloTag ligand (Janelia Fluor (JF) 549 or 646) to label HaloTag-linked PSD95 protein enables triple-color fluorescent labeling of cellular context, presynapse and postsynapse. All subsequent live-cell analysis was performed on cultures DIV1-2. **(D,E)** The resulting live triple-color labeling described in **(C)**, for the mouse lines Ai32-VC-KI-PSD **(D)** and Ai14-Neurog1-PSD **(E)**, respectively P6DIV2 **(D)**, P7DIV1 **(E)**. **(F,G)** Virally overexpressed RIBEYE-xFP colocalizes with presynaptic proteins Piccolino **(F)** and Bassoon **(G)**, P6DIV2; z-projections. **(F’, F”)** Isolated fluorescence of Piccolino **(F’)** and of RIBEYE-GFP **(F”)**, single plane of **(F)**. **(G’, G”)** Isolated fluorescence of RIBEYE-tdTomato **(G’)** and Bassoon **(G”)**, single plane of **(G)**. **(H)** Application of PSD95-HaloTag ligands **(H’)** results in a labeling pattern highly comparable to PSD95 antibody staining **(H”)**, showing almost complete colocalization, especially in basolateral IHC compartments. P6DIV1. **(I)** The PSD95-HaloTag mediated labeling results in a near-native fluorescence pattern of postsynaptic PSD95 partnering presynaptic ribbon precursors. P6DIV1. Scale bars: **(A, B)** left: 100 μm; middle: 50 μm; right: 10 μm; **(D–I)** 5 μm. Please also note the associated Supplementary Figures S1 and S2 as well as Supplementary Table S1.

Organotypic cultures of the organ of Corti were prepared from neonatal mice (P5-P7), of WT, Ai14-Neurog1-PSD, and Ai32-VC-KI-PSD background (Voorn et al., 2024) (Figure 1). Apical-medial turns of the organ of Corti were dissected out of the mouse cochlea and mounted on either 1.5 thickness high-precision coverslips (within a 35 mm Petri dish) or glass bottom Petri dish inserts (P35G-1.5-14-C, MatTek), coated with CellTak (#354240, Corning, 1:8 solution in NaHCO3). Upon proper attachment, the organotypic cultures were submerged in 2 mL culturing medium and incubated at 37 °C, 5% CO_2_ for up to 2 days *in vitro* (DIV2).

### 2.3 Viral transduction

Viral transduction *in vivo* was conducted by injection of neonatal animals of the Ai14-Neurog1-PSD and Ai32-VC-KI-PSD background with an adeno-associated virus vectors (AAV) PHP.B/eB (full constructs consisting of: PHP.B_CMV-HBA-RIBEYE-GFP_WRPE_bGH or PHP. eB_CMV-HBA_RIBEYE-tdTomato_WPRE_ bGH) (Table 1), 1 day prior to culture preparation (P4-P6) as described previously (Rankovic et al., 2021; Voorn et al., 2024). Viral overexpression allowed for live-cell imaging of RIBEYE-GFP on DIV1, whereas RIBEYE-tdTomato required incubation until DIV2, likely due to a slower chromophore maturation (Shaner et al., 2004; Guerra et al., 2022). Viral injections were performed through the round window of the right ear under 5% isoflurane anesthesia (5% for induction, 2%–3% for maintenance). Analgesia consisted of subcutaneous injection of buprenorphine (0.1 mg/kg body weight, approximately 30 min before the surgery), locally applied xylocain (10 mg spray, during surgery), and subcutaneous injections of carprofen (5 mg/kg body weight, applied during as well as 1-day post-surgery). All virus injection experiments were performed in compliance with the national animal care guidelines and were approved by both the board for animal welfare of the University Medical Center Göttingen and the State Animal Welfare office of Lower Saxony (AZ19/3133).

### 2.4 Application of JaneliaFluor HaloTag ligands and pharmacological treatment

Fluorescent live-cell labeling of the postsynaptic density (PSD) was conducted in our PSD95-HaloTag knock-in mouse lines using either the JaneliaFluor HaloTag 549 (JF549, #GA1110, Janelia Fluor® HaloTag®) or the JaneliaFluor HaloTag 646 ligands (JF646, #GA1120, Janelia Fluor® HaloTag®)—final concentration: 200 nM. The JF549 ligand was used in dual-color experiments in combination with overexpression of RIBEYE-GFP; the JF646 ligand was used in the triple-color experimental setup, combined with either RIBEYE-GFP or RIBEYE-tdTomato. The organotypic cultures were incubated with the ligand for 1.5 h, after which the complete media was replaced in one washing step and the sample was incubated for 3–8 h (37 °C; 5% CO_2_) to wash out excess fluorophore, prior to imaging.

Pharmacological treatment with the L-type Ca_V_ antagonist Isradipine (#2004, Tocris) was performed by drop-wise bulk application of 100 μL culture media to a final concentration of 10 μM, in between time-lapse imaging sessions.

### 2.5 Long-term multi-color time-lapse imaging

Time-lapse live-cell imaging experiments were conducted at an inverted Nikon Eclipse Ti-E microscope equipped with a Yokogawa CSU-WI Spinning Disk setup and Andor laser system, under environmentally-controlled conditions (37 °C, 5% CO_2_, Okolab Bold Line Cage Incubator), at 60x water immersion, NA 1.20. To prevent immersion medium evaporation during long-term imaging, we used RI-matched silicon oil instead of water for objective immersion (Immersol W 2010, Ne 1.33). Three-dimensional regions of interest (ROI) were selected for high intensity PSD-HaloTag labeling, low to moderate RIBEYE-tdTomato or RIBEYE-GFP expression, and, when present, genetic fluorescence indicating healthy morphology of IHCs or SGNs. ROI dimensions were minimized, while including a margin for potential drift. This resulted in an axial range of approximately 29 μm and varying spatial dimension (15–30 μm x and/or y), adapted to tissue orientation. Multi-color 3D time-lapse imaging was performed at maximum acquisition speed (consecutive color acquisition), conducting continuous imaging for a period of 40–90 min. For dual-color experiments, time-lapse images were acquired at intervals of 45–60 s, whereas triple-color acquisition required 70–80 s per time frame. For dual-color experiments, laser excitation was used at 488 and 565 nm (20 ms and 40 ms). For triple-color experiments, excitation was dependent on the experimental setup and mouse line used: for experiments with the Ai14-Neurog1-PSD line, excitation of 488, 565 and 637 nm was used; in case of the Ai32-VC-KI-PSD line, excitation was used at 515, 565 and 637 nm (20 ms, 20 ms and 40 ms respectively, in both experimental setups). All imaging frames were averaged 4 times.

### 2.6 Immunohistochemistry of organotypically-cultured or acutely-dissected organs of Corti

Organotypic cultures were fixed in 4% formaldehyde (in PBS) for 15 min on ice, and thereafter stored in PBS at 4 °C. Fixed organs of Corti were permeabilized in PBS + 0.5% TritonX-100 for 30 min, then incubated in blocking buffer (PBS + 0.5% TritonX-100 + 10% normal goat serum) for 1 h at room temperature. Incubation with primary as well as secondary, or directly-conjugated antibodies was performed in blocking buffer over 2 h at room temperature, while protected from light. Used antibodies can be found in Table 1. Live-cell imaging samples on glass bottom dishes were enclosed with an additional coverslip, whereas samples on coverslips were mounted directly on glass slides, in both cases using ProLong Glass Antifade reagent (#P36984, Invitrogen).

### 2.7 Confocal and super-resolution STED microscopy of fixed samples

Immunostained samples were imaged using an Abberior Instruments Expert Line STED microscope–operated in confocal laser scanning mode, using either a 20x/NA 0.8 or 100x/NA 1.4 oil immersion objective. For super-resolution STED microscopy, samples were imaged in 2D STED mode at a 10 nm xy pixel size. A pulsed 775 nm laser was used for stimulated emission depletion, while using 561 nm and 640 nm laser lines for excitation.

### 2.8 Processing and analysis of confocal and STED images of fixed samples

Confocal images of fixed organs of Corti were analyzed for ribbon volume and postsynaptic attachment in IMARIS (9.6.1, Oxford Instruments), using the Surface rendering function for ribbons (0.054 surface detail, local background subtraction, 0.28 μm largest sphere diameter, 0.15 split surface seed points, 3 quality filter), and for the PSD (0.08 surface detail, local background subtraction, 0.6 μm largest sphere diameter, 3 quality filter). Based on their proximity to the PSD, ribbon precursors were either classified as synaptically-engaged (≤500 nm), or non-synaptic (>500 nm). Super-resolution STED images were processed for display in FIJI/ImageJ (2.3.0/1.53q) (Schneider et al., 2012) using the Gaussian Blur filter at σ 1.0.

### 2.9 Time-lapse image processing and analysis

Time-lapse images were post-processed in FIJI/ImageJ (2.3.0/1.53q) (Schneider et al., 2012) to correct for bleaching using the BleachCorrection plugin, simple ratio algorithm [CorrectBleach (Miura, 2020)]; different fluorophores were corrected separately. Thereafter, two consecutive drift corrections were performed in IMARIS (9.6.1, Oxford instruments); the first correction based on the surface rendering of the cellular context (IHC, SGN, or PSD), the second correction originating from ribbon precursor particle tracking using the Spots and lineage tracing algorithm.

The following surface rendering parameters were used: for SGNs (0.22 surface detail, local background subtraction, 0.5 μm largest sphere diameter, 10 quality filter, autoregression tracing, 1.5 μm maximum distance, 0 maximum time gap), IHCs (0.26 surface detail, local background subtraction, 0.2 μm largest sphere diameter, 10 quality filter, autoregression tracing, 1.5 μm maximum distance, 0 maximum time gap) and PSDs (0.16 surface detail, 165 local background subtraction, 0.5 μm largest sphere diameter, 10 quality filter, autoregression tracing, 1.5 μm maximum distance, 0 maximum time gap). When the PSD-HaloTag labeling was used in combination with genetic YFP IHC plasma membrane fluorescence (Ai32-VC-KI-PSD), the PSD surface rendering was filtered for proximity to the IHC surface; when the PSD-HaloTag label was used in combination with genetic tdTomato SGN cytosol fluorescence (Ai14-Neurog1-PSD), the rendered SGN surface was used as a reference for the PSD-HaloTag channel.

Ribbon precursors were detected with the IMARIS Spots function (0.5 seed point diameter; 1.0 PSF correction, background subtraction, 15 seed point quality threshold, 45 region border growing; lineage tracing, 1.5 μm maximum distance, 0 maximum time gap, tracks minimum length 3 frames). Particle tracking for the precursors was conducted semi-automatically, using the lineage tracing algorithm. The fusion and fission of precursor particles was then checked manually, and based on fluorescence intensity center and independent particle mobility, individual particles were added to or removed from the automatically rendered tracks.

Live ribbon precursor volumes were detected using the surface rendering function (0.1 surface detail, local background subtraction, 0.28 μm largest sphere diameter, 0.3 split surface seed points, 3 quality filter, filter closest distance to Spots = Ribbons). For pharmacologically-treated conditions, local background subtraction was set to 25.

Ribbon precursor spots as well as surfaces were classified based on the spot-to-surface or surface-to-surface distance calculation in IMARIS. The synaptically-engaged ribbon class included the spots and surfaces in close proximity to the rendered PSD surface (≤500 nm), whereas the non-synaptic ribbon precursor class concerned those located more distally from the PSD (>500 nm distance to the rendered PSD surface). In context of the IHC membrane, non-synaptic ribbons were subdivided into membrane-proximal precursors (≤500 nm from the rendered IHC membrane surface) or free-floating, cytosolic precursors (>500 nm distance to the IHC membrane). Ribbon precursor classes were assigned per time frame, upon which all individual precursor data points over time were pooled per class. The classification of precursor tracks was assigned according to the predominant localization of precursors within the total trajectory: i) tracks containing synaptically-engaged ribbon precursors in minimally three consecutive imaging frames were classified as “synapse-interacting” or “synaptic” tracks; the non-synaptic tracks could, in context of the IHC membrane, be divided in two subcategories: ii) membrane associated tracks consisted of predominantly membrane-proximal ribbon precursors, whereas iii) cytosolic tracks exclusively contained precursors that resided in the cytosol or localized in IHC membrane proximity for less than three consecutive frames. Ribbon precursor surfaces, spots and tracks were controlled for atypical characteristics and intracellular localization, such as large protein aggregates or potentially disrupted tissue, and excluded from the analysis.

Track segments for the synaptic context to ribbon mobility correlation analysis were selected manually from the semi-automatically rendered traces, after removal of fusion and fission events. Here, segment selection was based on ribbon precursor location in respect to the rendered PSD surface, bordered by the fluorescent SGN: (i) segments classified as AZ-approaching contained precursors initially distanced from the PSD and a final three consecutive imaging frames localizing in engagement with the AZ; (ii) continuously synaptic segments excluded any time precursors spent outside of synaptic engagement of over three consecutive imaging frames; (iii) segments of ribbon precursors distancing from the AZ were required to contain initial localization in an synaptically engaged state of three imaging frames, after which precursors localized at a further distance from the labeled PSD. Importantly, the precursor location in the analyzed track segments was controlled for proximity to other PSD-HaloTag fluorescent clusters in addition to target PSD, to exclude for potential confounding factors for tracks interacting with multiple PSDs.

The assessment of three-dimensional ribbon precursor displacement within the track segments was performed as in Voorn et al. (2024). The mean squared displacement (MSD) was calculated based on the extracted *xyzt* ribbon precursor coordinates, rendered in the IMARIS Spots function by center of mass. The traversed displacement was then averaged over the progressive imaging frames (τ).

$$MSD\left(n\times \Delta t\right)=\frac{1}{N-n}\sum_{i=1}^{N-n}\left({\left[X_{i+n}-X_{i}\right]}^{2}+{\left[Y_{i+n}-Y_{i}\right]}^{2}+{\left[Z_{i+n}-Z_{i}\right]}^{2}\right)$$

Here, x, y and z are the ribbon precursor coordinates, N is the number of data points in the track segment, and Δτ is the time interval per imaging frame. The MSD curve was then fitted using least squares fit to determine exponent α, as well as parameter K.

$${MSD}_{fit}\left(\Delta t\right)=K\Delta \tau^{\alpha }$$

Track segment selection for ribbon precursors undergoing intersynaptic exchange was based on precursor location in respect to two different PSDs. Here, positional engagement was required with the primary AZ for three initial imaging frames, as well as thereafter with a secondary AZ for minimally three imaging frames. In cases of the two PSDs neighboring one another in such manner that the precursor trajectory remained in proximity to both PSDs while traversing, the length of the track segment was based on the relative distances between precursor and PSDs. From these segments, values such as displacement length, duration, mean and maximum velocity were extracted. For determination of precursors fusion at the secondary AZ, the subsequent continued track was also taken into account.

The two PSDs involved in intersynaptic exchange were identified based on cohesive HaloTag fluorescence and respective mobility. Here, the volume, area and dynamics of singular PSDs within clearly defined fluorescent SGN boutons were exemplary in identifying a selection of smaller PSD puncta in close proximity as one postsynaptic contact. In instances where this assignment proved difficult, the total was regarded as one postsynaptic contact.

The intracellular location of ribbon precursors was classified as pillar or modiolar according to assigned coordinates by placement of an axis origin point at the IHC base in IMARIS (“Reference Frame”). Based on the IHC orientation, pillar and modiolar sides could be distinguished. The axes of the Reference Frame were then positioned such that the x-axis was parallel to the line of neighboring IHCs and the z-axis was parallel to the positioning of the IHC *in situ*. For individual precursors, exact assigned coordinates were used for the analysis; for precursor tracks, the mean position over time was used.

### 2.10 Statistics

Statistical analyses were performed in Prism8 and 10 (GraphPad, San Diego, CA). Normality of the distributions was determined using a D'Agostino-Pearson test. The statistical tests on the time-lapse images were either done on individual precursor spots, traced ribbon precursor trajectories, or values per IHC.

**FIGURE 2:**
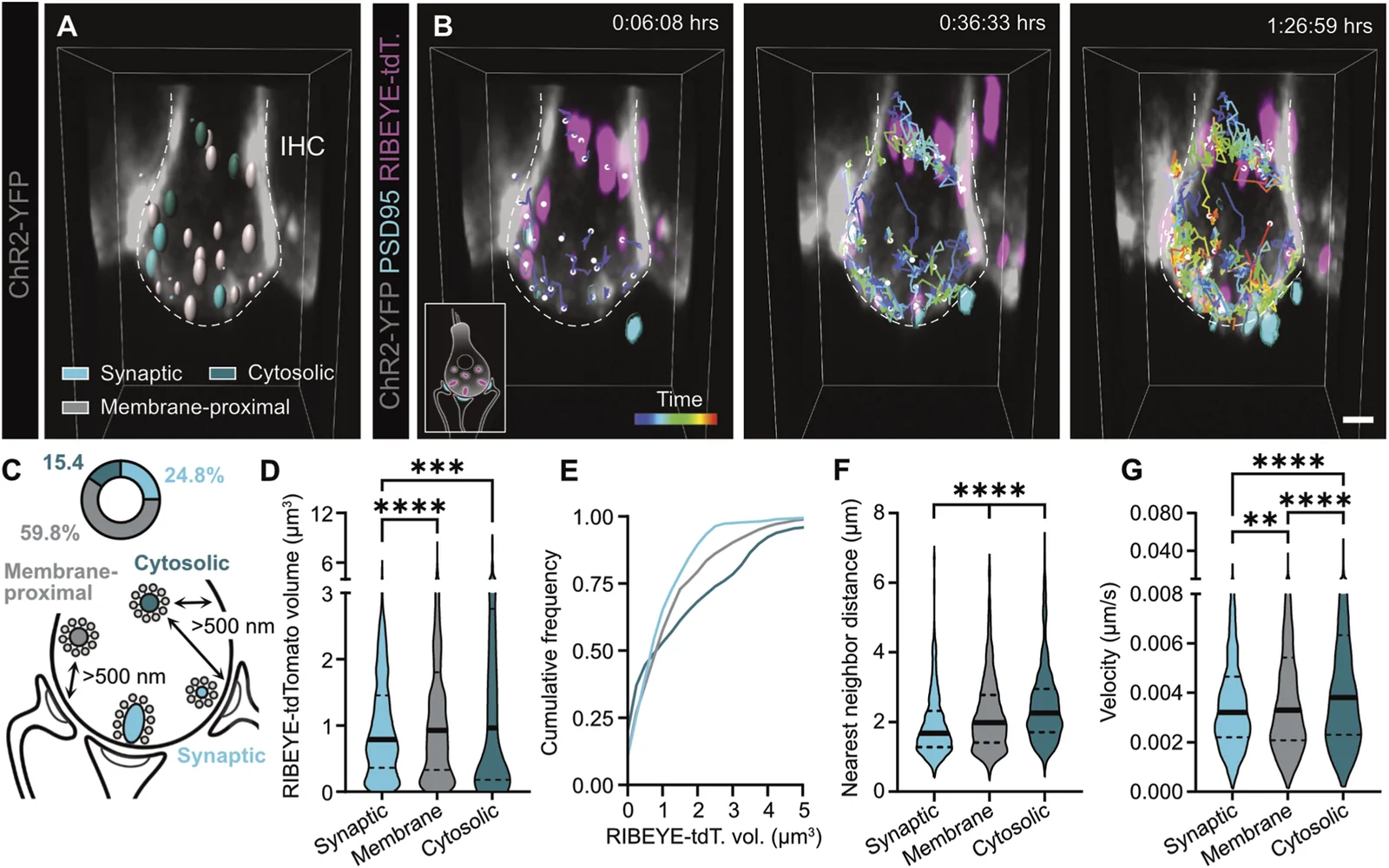
Ribbon precursor dynamics depend on the intracellular localization. **(A)** Representative 3D image of an IHC expressing RIBEYE-tdTomato. Ribbon precursors are indicated with spheres, color-coded for three different precursor classes based on their intracellular localization: synaptic, membraneproximal, and cytosolic. **(B)** Time-lapse imaging frames taken over 90 min, showing the progressive development and complexity of the ribbon precursor tracks, color-coded for time. The insert illustrates the experimental setup. Frames taken from a triple-color live imaged organotypic culture of the organ of Corti (P5DIV2), with endogenous IHC membrane fluorescence, overexpression of RIBEYE-tdTomato, and the PSD labeled by PSD95-HaloTag ligand (JF646). **(C)** Graphical illustration defining the three precursor classes based on a 500 nm distance cutoff from either the synaptic contact or IHC membrane. **(D)** The volume of synaptic ribbon precursors is lower than of membrane-proximal and cytosolic precursors. **(E)** The frequency distribution of the different precursor classes shows a significant fraction of the cytosolic precursors consists of considerably larger precursors. **(F)** The nearest neighbor distance (NND) of synaptic precursors is relatively low, while that of membrane-proximal precursors is slightly higher, and the NND of cytosolic precursors is much higher, indicating a more dispersed localization. **(G)** The velocity of synaptic ribbon precursors is slightly lower than of membrane-proximal precursors, whereas cytosolic precursors appear to displace at a much higher pace. **p < 0.01, ***p < 0.001, ****p < 0.0001. Statistical significance: Kruskal–Wallis. Nanimals = 4, nIHC = 9, ntraces = 179, nribbons = 10855 per 461 time frames (this equates to on average 23.5 ribbons/frame). Scale bar: 2 μm. Please also note the associated Supplementary Figure S3.

For Figure 2, ribbon precursors were characterized based on their intracellular environment by the extraction of values for each time frame and each individual rendered spot and surface separately. Here, ribbon precursors traced along one trajectory could be classified as synaptically-engaged in one frame (when in vicinity of the PSD), while being part of the membrane-proximal or cytosolic population in the next. All time point values were then pooled per ribbon precursor class. To determine the distribution of the different ribbon precursor classes, the sum of all ribbon precursors across all time points was used. To compare the different classes, Kruskal–Wallis tests were performed in combination with multiple comparisons Dunn’s *post hoc* tests.

**FIGURE 3:**
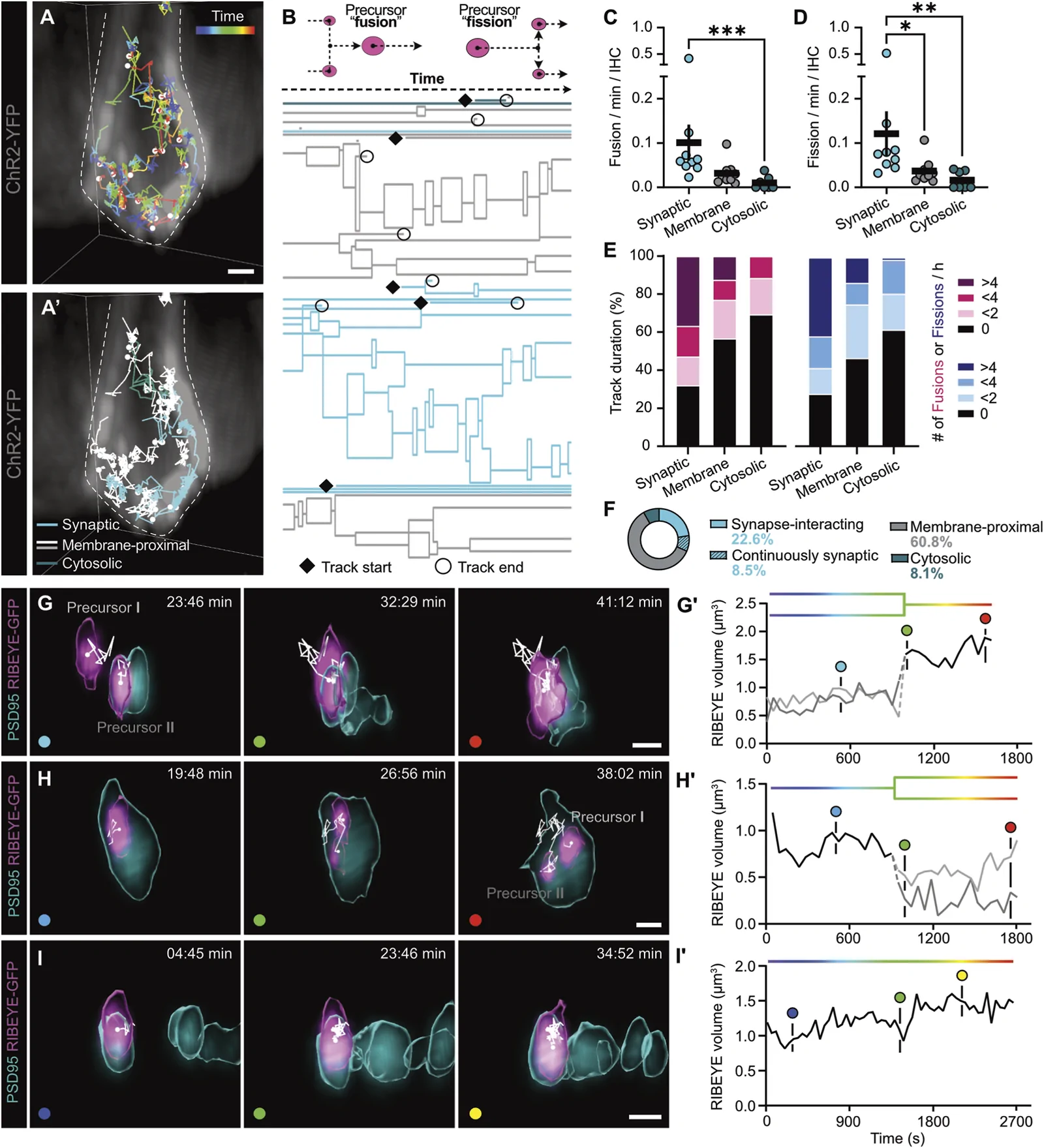
Ribbon precursor plasticity centers around the presynaptic AZ. **(A)** Exemplary 3D live imaged frame of an IHC expressing RIBEYE-tdTomato (P6DIV2), with indicated ribbon precursor tracks over 90 min, color-coded for time **(A)**, and for the three different precursor track classes **(A’)**: synapse-interacting, membrane-proximal and cytosolic. **(B)** Lineage tracing graph illustrating ribbon precursor plasticity over time. Each line represents a single precursor; mergers and splits display fusion and fission events. Certain precursor tracks appear to start during imaging time (diamonds), whereas other tracks become undetectable (circles). **(C,D)** Quantification of the plasticity event frequency shows that synapse-interacting precursor tracks contain more fusion events **(C)** than cytosolic tracks, and more fission events **(D)** than membrane-proximal, as well as cytosolic precursor tracks. **(E)** The frequency distributions of precursor plasticity demonstrate the synapse-interacting tracks to be highly plastic. **(F)** Distribution of the three different precursor track classes; synapse-interacting (31.1%; 39 h 17 min 53 s), membrane-proximal (60.8%; 76 h 59 min 35 s), and cytosolic (8.1%; 10 h 15 min 7 s), according to the total sum of track duration. A small subpopulation of the synapse-interacting tracks consists exclusively of ribbon precursors that continuously localize at the presynaptic AZ (of the total: 8.5%; 10 h 47 min 25 s; other synaptic fraction: 22.6%; 28 h 30 min 28 s). **(G–I)** Exemplary 3D rendered time-lapse imaging frames, showing volume adaptation of ribbon precursors at the AZ during fusion **(G)**, fission **(H)**, or stable, non-plastic localization **(I)** (IHC synapses imaged at P7DIV1). For videos of the examples, please refer to Supplementary Videos S4–S9. **(G’–I’)** Volume progression over time of the precursors in **(G–I)**, color coding as indicated in **(G–I)**. Time points of snapshots highlighted. Lineage tracing of precursor plasticity is indicated at the top of the graph, color-coded for time. *p < 0.05, **p < 0.01, ***p < 0.001. Statistical significance: Kruskal–Wallis. N_animals_ = 4, n_IHC_ = 9, n_traces_ = 179, n_ribbons_ = 10855 per 461 time frames (on average 23.5 ribbons/frame). Scale bars: **(A)** 3 μm; **(G–I)** 1 μm.

For Figure 3, full length ribbon precursor trajectories were taken into account to analyze precursor dynamics over time. The frequency of plasticity events was calculated per IHC for each track class, based on the total number of plasticity events and the sum of the duration of all track per class (for classification, see Section 2.9). Again, classes were compared using a Kruskal–Wallis test with *post hoc* Dunn’s multiple comparisons correction. The plasticity distribution per class was based on the track duration and number of events of all cells per class combined. For the overall distribution of track classifications, the total track duration sum was used.

**FIGURE 4:**
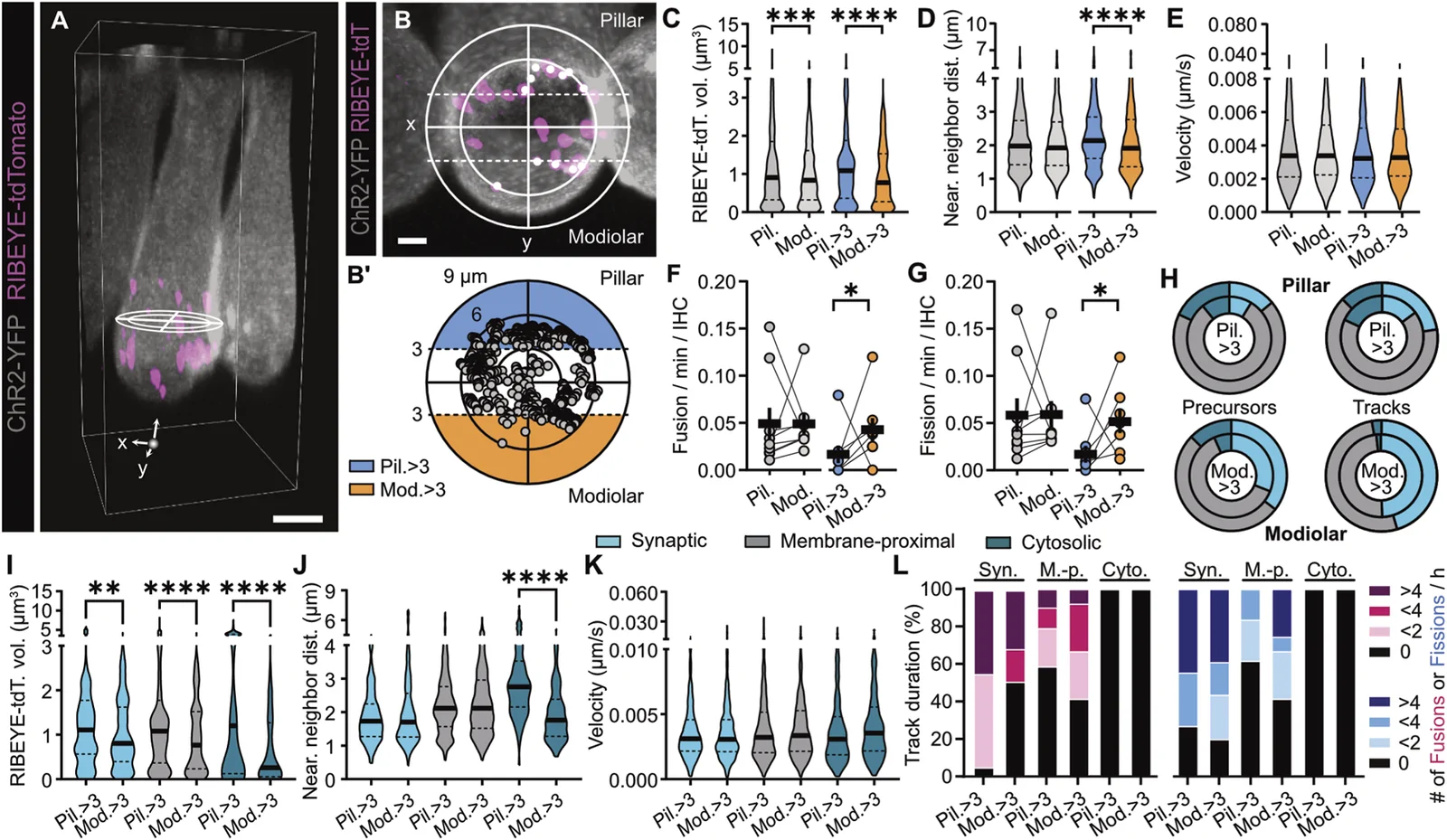
Ribbon precursor plasticity differs along the pillar/modiolar IHC axis. **(A)** Exemplary 3D live image of an IHC expressing RIBEYE-tdTomato, with x- and y-axes indicated used to determine pillar and modiolar IHC fractions (P7DIV2). **(B)** Top-down view of **(A)**. Dotted lines demonstrate the pillar/modiolar extremities of the IHC >3 μm from the 0 midline. **(B’)** Illustration of the >3 μm extremities, displaying all precursors over time. **(C–G)** Pillar/modiolar populations based on the 0 midline (grays), and the >3 μm division (blue/orange). **(C)** The volume of modiolar precursors is lower than of pillar precursors. **(D)** In the >3 μm extremities, the nearest neighbor distance (NND) is lower for modiolar than pillar precursors. **(E)** Ribbon precursor velocity is comparable throughout the IHC. **(F, G)** The frequency of fusion **(F)** and fission **(G)** events is increased in modiolar tracks in the >3 μm extremities. **(H)** The percentage of synaptically-engaged precursors and tracks is considerably higher in the modiolar than the pillar IHC fraction. The distribution of the total (outer ring) precursors: pil. synaptic 14.0%, pil. membrane 68.0%, pil. cytosolic 18.0%; mod. synaptic 35.5%, mod. membrane 51.8%, mod. cytosolic 12.7%; and tracks: pil. synaptic 22.4% (15 h 5 min 16 s), pil. membrane 65.0% (43 h 40 min 44 s), pil. cytosolic 12.6% (8 h 27 min 59 s); mod. synaptic 45.4% (26 h 56 min 59 s), mod. membrane 51.8% (30 h 42 min 32 s), mod. cytosolic 2.8%(1 h 39 min 4 s). And the distribution of the >3 μm precursor population (inner ring): pil. synaptic 8.7%, pil. membrane 80.4%, pil. cytosolic 10.8%; mod. synaptic 31.1%, mod. membrane 62.2%, mod. cytosolic 6.7%; and >3 μm precursor tracks: pil. synaptic 14.9% (3 h 4 min 46 s), pil. membrane 67.0% (13 h 53 min 21 s), pil. cytosolic 18.1% (3 h 45 min 5 s); mod. synaptic 49.5% (8 h 34 min 31 s), mod. membrane 48.8% (8 h 27 min 40 s), mod. cytosolic 1.7% (18 min 9 s). **(I–K)** Displaying solely the >3 μm populations. **(I)** The volume of modiolar precursors was lower than of pillar precursors in all precursor classes. **(J)** The lower modiolar NND appears to be driven by the cytosolic precursors. **(K)** Precursor velocity was similar for all classes. **(L)** The plasticity distribution shows a slightly higher frequency of fission events in modiolar synaptic tracks. *p < 0.05, **p < 0.01, ***p < 0.001, ****p < 0.0001. Statistical significance: Kruskal–Wallis. N_animals_ = 4, n_IHC_ = 9, Total: n_traces_ = 179, n_ribbons_ = 10855 per 461 time frames (on average 23.5 ribbons/frame); >3 μm populations: n_traces_ = 55, n_ribbons_ = 5557 per 461 time frames (on average 12.1 ribbons/frame). Scale bars: **(A)** 5 μm; **(B)** 2 μm. Please also note the associated Supplementary Figure S4.

For the analysis of pillar/modiolar heterogeneity in ribbon precursor dynamics in Figure 4, individual ribbon precursor spots and surfaces were charted along the pillar/modiolar axis per time frame separately. The frequency of plasticity events, and overall class distribution was determined as described for Figure 3, based on the mean localization of the track along the pillar/modiolar axis. When comparing pillar and modiolar populations, statistical significance between the two groups was determined with a Mann-Whitney U test. When comparing classes, a Kruskal–Wallis test was performed, with a Dunn’s multiple comparisons correction between the pillar/modiolar partner populations for each class.

**FIGURE 5:**
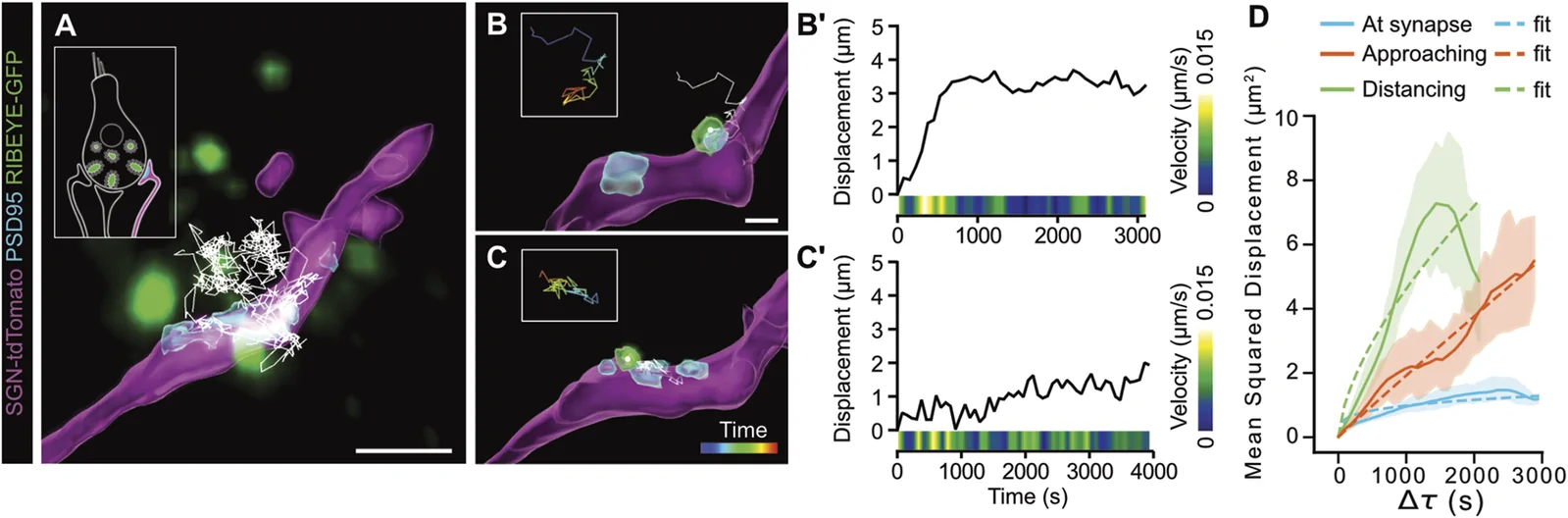
The context of the presynaptic AZ confines ribbon precursor mobility. **(A)** Exemplary 3D reconstruction of a tdTomato-positive SGN with the PSD labeled by PSD95-HaloTag ligand (JF646), rendered from a live triple-color imaged organotypic culture of the organ of Corti (P6DIV1). The SGN innervates a RIBEYE-GFP expressing IHC, of which the traced ribbon precursor trajectories are indicated that interact with the synaptic contact of the SGN over 90 min of time-lapse imaging. The insert illustrates the experimental setup. **(B)** Trajectory segment of a ribbon precursor that has displaced/traversed in approaching motion towards the SGN, and now localizes apposed a PSD inside the SGN. **(C)** Trajectory segment of a ribbon precursor continuously localized in engagement with the PSD inside the SGN, therefore likely continuously at the AZ. **(B, C)** Inserts: trajectory segment color-coded for time. **(B’, C’)** The displacement length and velocity (color-coded) of the ribbon precursors in examples **(B)** and **(C)** over time, showing a steep rise in displacement and peak in velocity when **(B’)** the ribbon precursor approaches the synapse. **(D)** The mean squared displacement (MSD) of three different trajectory segment classes: precursors in motion of AZ approach (orange), distancing from the AZ (green) and in continuous association with the AZ (cyan). The displacement of precursors at the AZ is confined compared to more mobile precursors in distancing or approaching motion. At synapse, α = 0.24; Distancing, α = 0.64; Approaching, α = 0.97. N_animals_ = 7, n_IHCs_ = 12, n_segments_ = 18. Scale bars: **(A)** 5 μm; **(B,C)** 1 μm.

**FIGURE 6:**
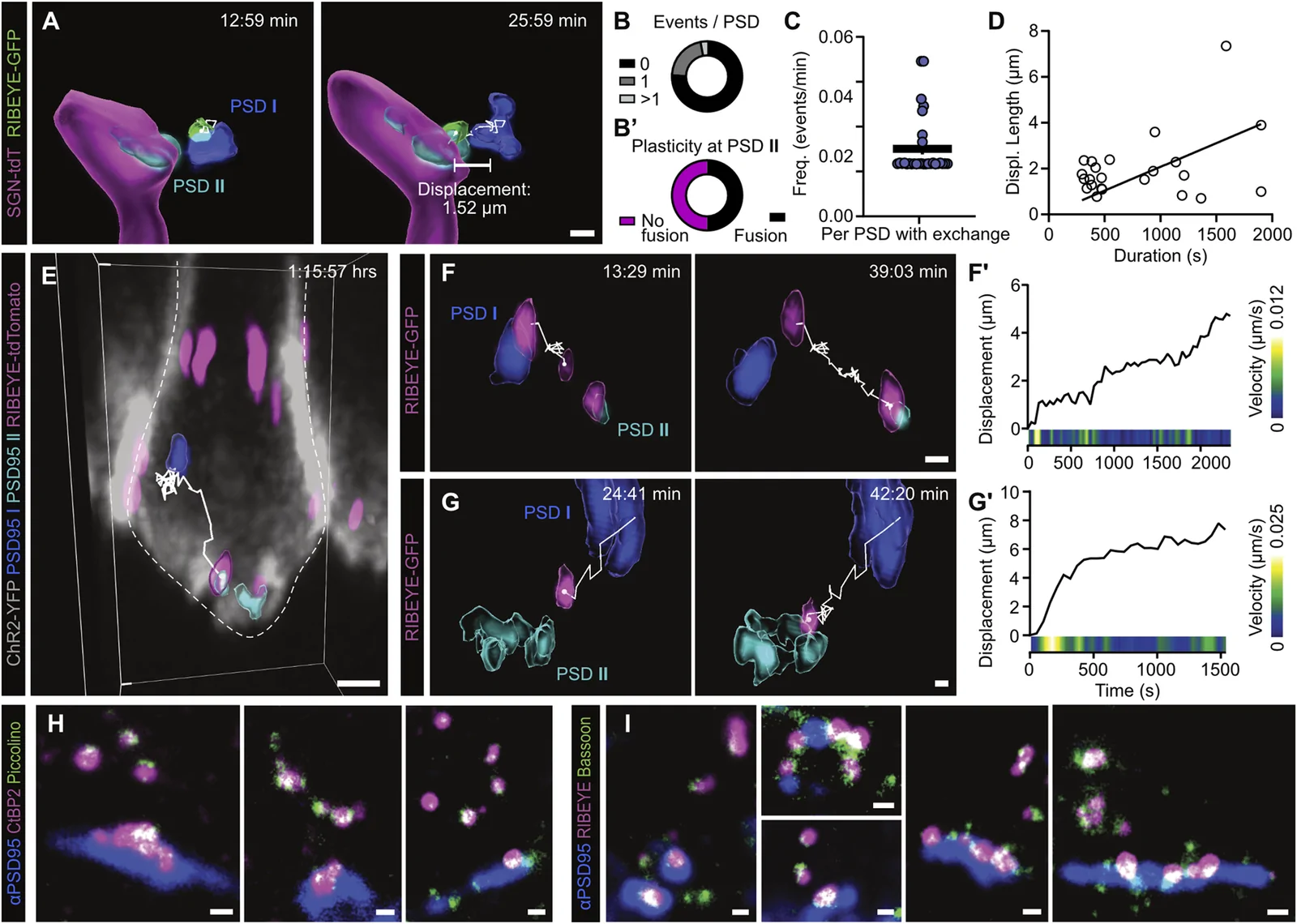
Intersynaptic exchange of ribbon precursors takes place between different presynaptic AZs. **(A)** Illustrative example of intersynaptic exchange between a primary synaptic contact (dark blue PSD) situated outside and a secondary synapse (cyan) inside the fluorescent SGN; rendered from a triple-color live imaged organotypic culture of the organ of Corti (P7DIV1). **(B)** Frequency of events of intersynaptic exchange per traced postsynapse. **(B’)** Frequency of ribbon precursor fusion at the secondary synapse. **(C)** Frequency of events per PSD for which intersynaptic exchange was detected. **(D)** The displacement length of intersynaptic exchange trajectories showed a positive, but non-significant correlation with the trajectory duration. Y = 0.002198*X, p = 0.6769. **(E)** Example of traced intersynaptic exchange of a ribbon precursor along the IHC membrane (IHC imaged at P5DIV2). **(F)** Example of intersynaptic exchange where a small precursor can be observed to bud off from the larger precursor engaged at the primary synapse. During the exchange, the synaptically-engaged precursor uncouples from the primary PSD (IHC synapse imaged at P5DIV2). **(G)** Example of intersynaptic exchange of a precursor traversing a long distance between the two PSDs, with an initial rapid distancing from the primary synaptic contact (IHC synapse imaged at P7DIV1). **(F’, G’)** Displacement length and precursor velocity (color-coded) over time of examples in **(F)** and **(G)**. **(H,I)** STED microscopic images of synaptically-engaged and non-synaptic precursors, showing colocalization of **(H)** RIBEYE (labeled by CtBP2) with Piccolino in precursors in both intracellular locations, as well as **(I)** colocalization between RIBEYE and Bassoon. N_animals_ = 10, n_IHC_ = 14, n_traces_ = 23. Scale bars: **(A,F,G)** 1 μm; **(E)** 2 μm; **(H,I)** 500 nm.

**FIGURE 7:**
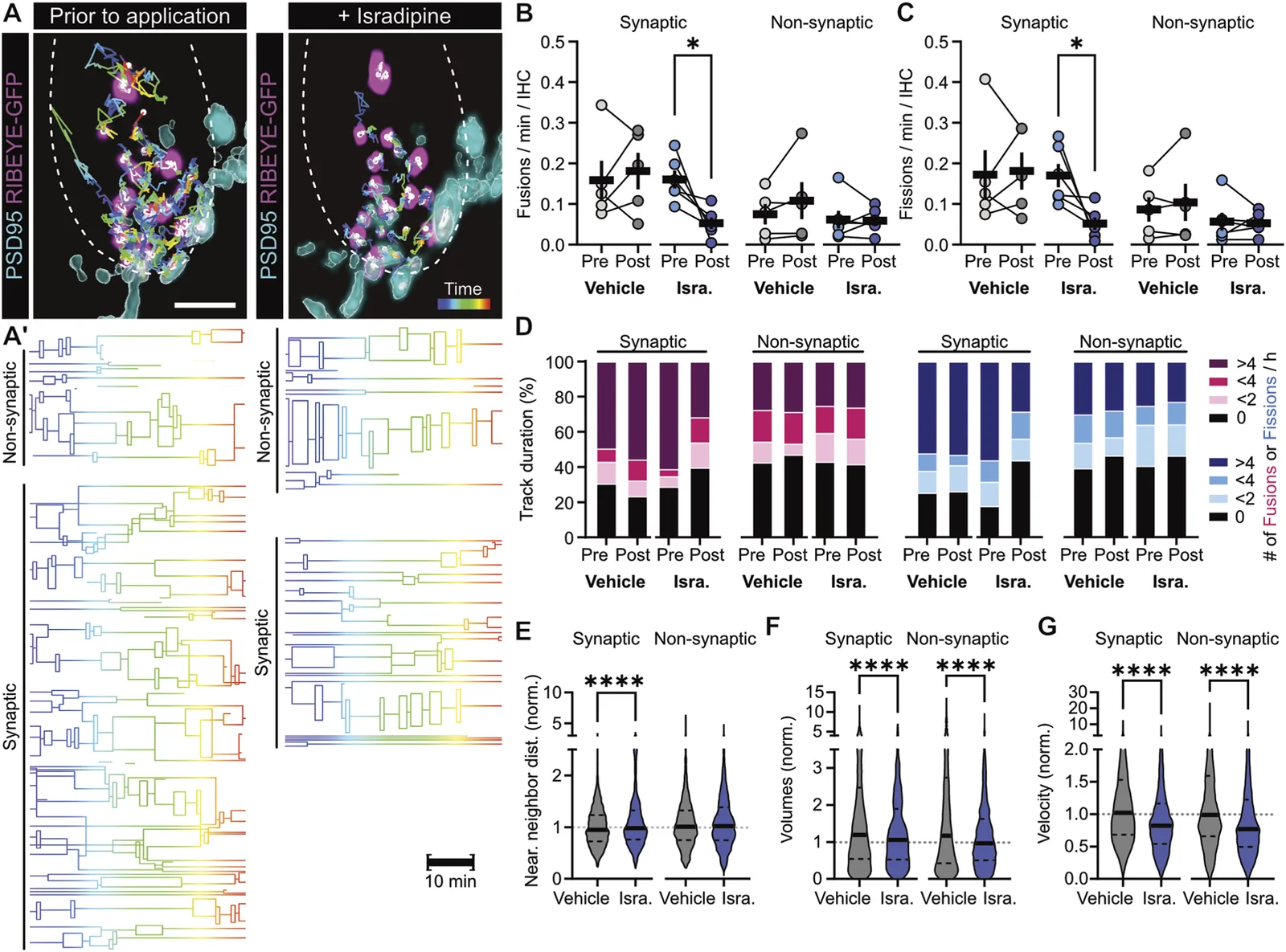
Inhibition of presynaptic Ca^2+^ influx reduces ribbon precursors plasticity around the AZ. **(A)** Exemplary ribbon precursor traces over 45 min of an IHC expressing RIBEYE-GFP prior and post Isradipine application; PSDs are labeled by PSD95-HaloTag ligand (JF549), rendered in 3D from a live dual-color imaged organotypic culture of the organ of Corti (P7DIV1). **(A’)** Lineage tracing graphs prior and post Isradipine application show the dramatic reduction in complexity of the synaptic precursor traces in A, while the number of tracks did not change significantly (mean pre Isra. 9.33 ± 2.4 vs. post 8.33 ± 1.52, p > 0.999). **(B,C)** The frequency of fusions **(B)** and fissions **(C)** upon Isradipine treatment is reduced in synaptic precursor tracks, whereas non-synaptic tracks are unaffected. Values displayed are pre and post pharmacological application, compared to a vehicle control (DMSO). **(D)** The frequency distribution of plasticity events shows that the profile of synaptic tracks is reduced to the level of non-synaptic tracks upon exposure to Isradipine, for both fusion events (magenta) and fission events (blue). **(E)** The nearest neighbor distance of ribbon precursors at the synapse is unaffected by Isradipine, but is slightly decreased in control conditions. **(F)** A small increase in precursor volume is present in the control condition, while this is less prominent in synaptically-engaged precursors, and absent in non-synaptic precursors in the Isradipine treated condition. **(G)** Isradipine application reduced ribbon precursor velocity, whereas the control condition remained unchanged. **(D,E,F)** Values displayed are post pharmacological treatment, normalized against the median of the paired precursor class prior to treatment. ****p < 0.0001. Statistical significance: Kruskal–Wallis. Nanimals = 7, nIHC = 11, ntraces_pre = 224, ntraces_post = 235, nribbons_pre = 22198, per 613 time frames, nribbons_post = 20258, per 613 frames (this equates to on average 36.2 ribbons/frame (pre) and 33.0 ribbons/frame (post)). Scale bar: 5 μm. Please also note the associated (Supplementary Figures S5 and S6).

For the specific detected behavior of ribbon precursor subpopulations in Figures 6, 7, such as synapse-approaching or undergoing intersynaptic exchange, trajectory segments were selected that spanned the observed ribbon dynamics. Correlation between various parameters was determined using the Pearson’s R correlation matrix.

For the pharmacologically-treated conditions in Figure 7, the frequency of plasticity events was calculated as described above and analyzed with Kruskal–Wallis tests, followed by a Dunn’s multiple comparison test between pre- and post-treatment application values of the same class, as well as between pre values of the treated and non-treated conditions of the same class. For the quantification of individual ribbon precursor parameters, data were extracted as described for Figure 2, upon which post-treatment values were normalized against the median of the matching classification pre-treatment (e.g., non-synaptic ribbon precursors pre- and post-treatment). In the graphical representation, post-treatment values only are represented. Kruskal–Wallis tests with *post hoc* Dunn’s multiple comparisons corrections were used to compare between treatment conditions.

## 3 Results

### 3.1 Monitoring real-time ribbon dynamics *in situ*

To investigate the mobility and structural plasticity of IHC ribbon precursors, we established triple-color live-cell synapse labeling by crossbreeding various reporter mouse lines for cellular contexting of either the IHC plasma membrane—via YFP-tagged ChR2 expression in IHCs (Ai32-VC-KI-PSD)- or the postsynaptic terminal, through cytosolic tdTomato expression in a small percentage of SGNs (Ai14-Neurog1-PSD) (Figures 1A,B). We then performed intra-cochlear AAV injections to introduce spectrally-matched RIBEYE-xFP and acutely applied fluorescent HaloTag ligands to visualize PSD95 (Figures 1C–E). Since continued viral overexpression may cause experimental artifacts, we opted for a short-term culture system, in which transduced IHCs were analyzed within 2–3 days post-injection and did not remain in culture for longer than 2 days upon dissection. Here, ribbon counts and volumes were indistinguishable from wildtype ribbons (Voorn et al., 2024), and RIBEYE-xFP colocalized with the presynaptic proteins Piccolino and Bassoon (Figures 1F,G). We then evaluated the labeling efficiency of different HaloTag ligands (JF646 and JF549) using *post hoc* immuno-analysis with a PSD95-directed nanobody. Both ligands accurately labeled the PSD and displayed excellent colocalization with αPSD95 staining around the basolateral IHC compartment (Figure 1H), where Halo ligand fluorescence partnered with endogenous ribbons (Figure 1I).

For the subsequent time-lapse imaging experiments to track ribbon dynamics, IHCs were selected based on morphological cell health and moderate RIBEYE-xFP expression levels. Here, a *post hoc* correlation analysis assisted in controlling for the effect of the varying expression levels per IHC on the extracted parameters (Supplementary Table S1). Importantly, while we had to pool data from a limited postnatal age range (P5-7) and subsequent time in culture (DIV1-2) for logistical reasons, direct comparison of various mobility-related parameters between the different RIBEYE-xFP variants as well as assessment of the individual time in culture did not reveal any systematic differences between the experimental cohorts (Supplementary Figure S1). Moreover, the ribbon volumes of organotypically-cultured IHCs were indistinguishable from age-matched acutely dissected preparations after fixation (Supplementary Figures S2A–D).

### 3.2 IHC ribbon synapse development is a dynamic process

Upon successful validation, we first studied IHC ribbon precursor dynamics in different intracellular fractions by longitudinally tracking tdTomato-labeled ribbon precursors within the basolateral IHC compartment. Due to the structural contexting, we could distinguish three distinct classes of ribbon precursors: (i) synaptically-engaged precursors, (ii) membrane-proximal, but non-synaptic precursors, and (iii) cytosolic precursors (Figure 2A), which is in line with the differential types identified in early electron microscope studies (Sobkowicz et al., 1986). These classes could then be differentially analyzed for their respective mobility behavior (Figure 2B; for the tracing of ribbon dynamics, see also Supplementary Videos S2, S3). Of the total precursor population, the majority registered as non-synaptic/membrane-proximal (∼60%), whereas ∼25% were synaptically-engaged, and another ∼15% cytoplasmically-floating (Figure 2C). While the observed fraction of non-synaptically-engaged ribbon precursors may appear surprisingly high and could be suspected to result from excessive synapse loss resulting from dissection- or culture-related degeneration of postsynaptic SGNs (Huang et al., 2007; Barclay et al., 2011), direct comparison with ribbon counts from age-matched acutely dissected organs of Corti and previously reported numbers only indicated a notable increase in the cytosolic precursor population (Supplementary Figures S2E, S2F) (Liberman and Liberman, 2016; Stalmann et al., 2021).

Subsequent volume analysis of the live-cell data revealed that the detected synaptic precursors were on average smaller than non-synaptic/membrane-proximal or cytosolic precursors (Figures 2D,E). Yet, they formed denser clusters than the membrane-proximal precursors, whereas cytosolic precursors were considerably more dispersed (Figure 2F). Next, we examined the dynamic characteristics of ribbon precursors across the acquisition time and found that cytosolic precursors displaced with the highest velocity, while membrane-proximal and synaptically-engaged precursors displayed increasing degrees of confinement (Figure 2G). Importantly, the observed trends for the nearest neighbor distance (NND) and velocity were maintained in cell averaged values (Supplementary Figure S3), confirming that, although small, these differences are present throughout the IHC population. Together, the crowding and reduced velocity at the presynaptic AZ is compatible with molecular trapping of ribbon precursors within the CAZ.

### 3.3 Ribbon precursor plasticity centers around the developing AZ

Consistent with our previous work (Voorn et al., 2024), long-term tracking of ribbon precursor dynamics yielded a complex network of interconnected trajectories that was characterized by numerous fusion and fission events (Figures 3A,B). Upon categorization, synaptic tracks appeared most complex and displayed the highest number of plasticity events of the different track classes (Figure 3C). However, surprisingly, fusion events were matched in frequency by fissions (i.e., plasticity events where a ribbon would *lose* material) thus rendering the bi-directional structural plasticity of ribbon precursors at nascent AZs strikingly balanced (Figure 3D). Moreover, when calculating the frequency distributions, synaptic tracks were most plastic and underwent several consecutive events of fission and re-fusion in our imaging window–apart from a small non-interactive subpopulation that solely underwent internal remodeling while continuously residing at the AZ (Figures 3E,F). While fusions clearly present a physiological mechanism of rapidly boosting ribbon volume (Figures 3G,G’), fissions likely serve to cap ribbon size and redistribute the material of an initially large ribbon precursor over multiple smaller secondary precursors—potentially with a secondary function in ‘seeding’ additional AZ scaffolds at novel locations (Figures 3H,H’). Consistent with previous work from zebrafish lateral line neuromast hair cells [nHC (Graydon et al., 2017)], we observed instantaneous appearances of up to that point likely diffraction-limited precursors in our observation volume indicative of *de novo* precursor assembly from a soluble RIBEYE pool. In further support of such a “cytoplasmic reservoir” hypothesis, we detected a gradual volume increase in synaptic ribbons lacking detectable plasticity events (Figures 3I,I’).

### 3.4 Synapse location influences ribbon precursor plasticity

Several studies have detailed the functional heterogeneity of synaptic contacts in mature IHCs. Here, location-dependent differences in synapse volume, voltage-dependence of Ca^2+^ influx, postsynaptic SGN caliber, excitability and RNA profiles give rise to a pillar/modiolar gradient of low to high threshold synapses (Ohn et al., 2016; Shrestha et al., 2018; Sun et al., 2018; Wang et al., 2021; De Faveri et al., 2025). While the postsynaptic diversification has been suggested to originate in the earliest developmental stages and requires pre-sensory SA, the mechanisms underlying presynaptic heterogeneity and its temporal sequence remain unclear. However, recent findings demonstrate early differentiation between low vs. high spontaneous rate (SR) SGN profiles along the pillar/modiolar IHC perimeter of young postnatal mice *in vivo* (De Faveri et al., 2025). We thus probed if location-dependent differences in ribbon dynamics could also be detected in our live-cell imaging paradigm and mapped individual ribbon precursor trajectories onto the pillar/modiolar-cuticular/habenular IHC axes (Figures 4A,B). We then performed location-based comparative analyses of all precursors, separated by a hard midline “0” cutoff between the pillar/modiolar IHC halves to observe global effects. To refine this analysis, we then excluded the subpopulation with intermediate coordinates and selected for the precursors at the pillar and modiolar “extremes,” which are poised to give a clearer picture on low-threshold, high SR vs. high-threshold, low SR fibers (Figure 4B’). Here, the global trends observed in the basic midline pillar/modiolar distinction were strikingly exacerbated in the selective populations, with the > ±3 μm cutoff offering the clearest representation of these effects without compromising the statistical power of our analysis (for additional cutoffs, please refer to Supplementary Figure S4). While individual ribbon volumes were lower on the modiolar side (Figure 4C), these ribbons clustered at a lower NND (Figure 4D). We then investigated the dynamic properties of ribbon precursors and, while we found no location-dependent variations in velocity (Figure 4E), the level of ribbon precursor plasticity significantly differed between the IHC extremes, as reflected by a higher frequency of fusion and fission events in the modiolar tracks (Figures 4F–H). Upon closer inspection of the precursor subpopulation characteristics, we additionally found that modiolar ribbon volumes were lower across all precursor classes and NND only differed between the cytosolic precursor fractions (Figures 4I,J). For completion, we also assessed precursor dynamics and found no difference in precursor velocity (Figure 4K), but a notable trend towards lower modiolar fusion frequency in synaptic tracks (Figure 4L).

Overall, these data suggest that certain location-dependent differences along the pillar-modiolar IHC axis can already be observed as early as the second postnatal week of development, which is notable in context of IHC planar synapse heterogeneity at later maturational stages.

### 3.5 Synaptic engagement restricts ribbon precursor mobility

We next shifted our focus from the entire basolateral IHC compartment to individual synaptic contacts and employed the Ai14-Neurog1-PSD line, in which sparse labeling of individual SGNs allowed us to trace ribbon dynamics specifically at the PSD of a singular afferent bouton (Figure 5A). We first traced all RIBEYE-GFP labeled precursors proximal to the fluorescent SGN, and then selected for AZ-interacting tracks. Intriguingly, individual ribbon precursors displayed various distinct mobility patterns along their trajectories (Figure 5B): for example, while the AZ approach often occurred with large displacement and high velocity, both parameters drastically decreased upon PSD engagement and continued residence at the AZ (Figure 5B’). Here, the apparent ‘capture’ of the precursor in the CAZ network did however not completely halt its mobility, but confined its movements (Figures 5C–C’).

To now differentially quantify the interplay between ribbon precursor mobility and AZ engagement, we further analyzed stable track segments and, based on their position, categorized them as: (i) AZ-approaching, (ii) continuously synaptic, or (iii) AZ-distancing. We then plotted the mean squared displacement (MSD) for the different track segments, and fitted the average per interaction type (Figure 5D). We found that displacement of the continuously synaptic precursors was strongly confined, which is compatible with a mechanism of diffusional trapping. On the other hand, the mobility of the AZ-approaching and AZ-distancing precursors—whether it was in a wandering or targeted motion—exhibited distinctly higher displacements.

### 3.6 Ribbon precursors undergo intersynaptic exchange between AZs

Since the sparse SGN labeling approach allowed us to delineate between PSD fragments within a given terminal and neighboring PSDs of nearby fibers (Figure 6A), we could analyze the frequency of intersynaptic exchange events. Here, we observed precursors to detach from established synaptic contacts, or to undergo fission events from synaptically-engaged ribbons that resulted in transfer of ribbon material to a secondary PSD. Although challenging to quantify, we estimated intersynaptic exchange to occur at ∼25% of all traced postsynaptic sites (Figures 6A,B). However, this might be a cautious estimate due to our conservative assignment of individual postsynaptic contacts in cases of more ambiguous identification without fluorescent SGN context label. Of the sites displaying intersynaptic exchange, the frequency of occurrence was generally low (Figure 6C), with mostly single plasticity events presenting during our observation window. In few cases, multiple events could be detected, where a single site could be both donating and accepting ribbon material, or have precursors detach and re-attach in the same location. In ∼50% of cases, intersynaptic exchange events were followed by fusion of the exchanged material with a membrane-resident synaptic ribbon at the secondary postsynapse (Figures 6B’,E,F; Supplementary Videos S10–S12). While the traveled distance and duration of exchange varied considerably between individual events, it displayed a positive–yet statistically non-significant—trend (Figure 6D). Notably, most of these events occurred between directly neighboring synapses and where hence short distanced; however, several instances covered multiple micrometers (range: 0.56–7.35 μm), with ribbon precursors translocating across the entire basolateral IHC pole in a surprisingly targeted manner that followed a near-linear trajectory (Figure 6E). Interestingly, we also observed instances of seemingly targeted slow precursor ‘walking’ motion or mixed modes (Figures 6F–G’). Regardless of the translocation mode, closer inspection of the velocity profiles of detaching precursors revealed an initial velocity peak upon detachment from the primary ribbon/AZ, indicative of an energy barrier that needed to be overcome for successful fission.

Since ribbon size has a direct impact on the SV tethering and Ca_V_ clustering capacity of a given AZ, we next employed STED nanoscopy to interrogate the molecular identity of AZ-proximal, cytoplasmically-floating ribbon precursors (Figures 6H,I). Consistent with previous reports (Regus-Leidig et al., 2013; Michanski et al., 2019), we identified the ribbon-specific protein Piccolino as an essential structural component, albeit at vastly varying quantities. Moreover, the ribbon-anchoring protein Bassoon consistently co-localized with synaptically-attached, but also free-floating precursors. This suggests that the dynamic plasticity of the maturing AZs involves the transfer of functional ribbon building blocks, which could rapidly—and reversibly—modify exocytic output of a given AZ.

### 3.7 Blocking presynaptic Ca^2+^ influx alters ribbon precursor structural plasticity

Lastly, we investigated the influence of pre-sensory SA on developmental ribbon dynamics. Considering the plastic changes in ribbon precursor size that have been described in young nHCs upon manipulation of presynaptic Ca^2+^ influx (Sheets et al., 2012), we suspected that pre-sensory SA may also play a regulatory role in mammalian auditory ribbon synapse development. Therefore, we utilized the intrinsic SA retained in organotypically-cultured organs of Corti (Tritsch et al., 2007; Zhang-Hooks et al., 2016; Halim et al., 2026) (see Supplementary Video S13), and first assessed if acute SV release bouts would trigger ribbon displacement due to excessive AZ membrane turnover. Here, we correlated ribbon dynamics with postsynaptic activation using GCaMP6-triggered averaging of velocity traces, but failed to detect reproducible patterns–thus suggesting statistical independence of these parameters (Supplementary Figure S5). We then pharmacologically blocked presynaptic Ca^2+^ influx with the L-type Ca_v_ channel antagonist isradipine—thereby effectively silencing IHC signal transmission—and performed sequential time-lapse imaging before and after isradipine or vehicle application (Figures 7A–C). Remarkably, isradipine strikingly reduced trajectorial complexity of synaptic tracks, without affecting the overall number of tracks. When quantifying the frequency of plasticity events, the occurrence of both, fusions and fissions, was significantly reduced upon isradipine exposure, whereas the vehicle group remained unaffected. Similarly, non-synaptic tracks displayed unaltered behavior, regardless of treatment. When plotting the frequency distributions of individual plasticity events (Figure 7D), fusions and fissions within the synaptic tracks were starkly reduced to a level comparable to non-synaptic tracks, hence indicating that presynaptic Ca^2+^ entry is indeed a strong driver of structural adaptation at IHC AZs.

To further extend this analysis, we examined the effects of isradipine on individual precursor level. We detected small changes in the NND and volume in untreated IHCs, which indicate a potential progression towards larger ribbons, and denser clustering at the synapse over time. Exposure to isradipine hence appears to hinder this progression, resulting in unchanged NND (Figure 7E) and a small but statistically significant volume increase (Figure 7F). However, when analyzing the cell average changes, these small trends were less clear (Supplementary Figures S6A, S6B) and therefore, the biological significance of these observations remains unclear at this point. On the other hand, isradipine treatment significantly reduced precursor velocity—for both, synaptic and non-synaptic precursors (Figure 7G)—whereas the velocity in control conditions remained unchanged—a finding that was also robustly represented in the population data (Supplementary Figure S6C). Together, these data suggest that the loss of presynaptic Ca^2+^ influx attenuates ribbon precursor mobility and plasticity around the presynaptic AZ, and may thereby play a prominent role in ribbon synapse development.

## 4 Discussion

**FIGURE 8:**
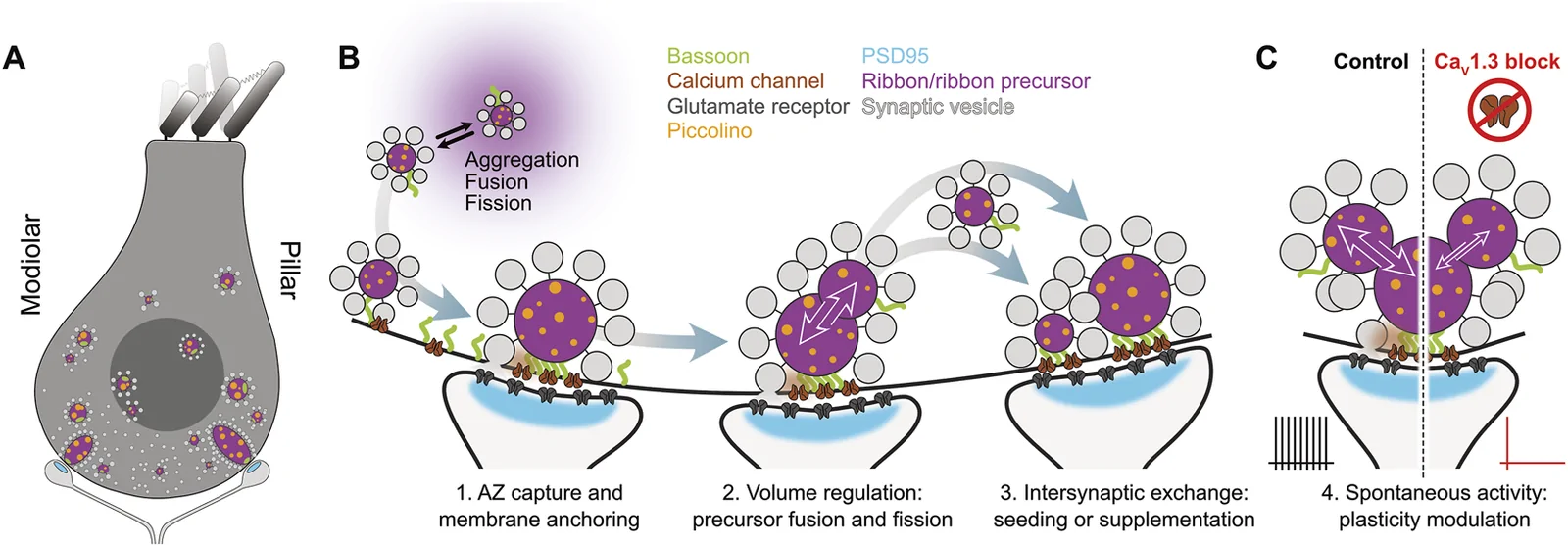
Overview of IHC ribbon synapse assembly in early postnatal development. **(A)** The structural plasticity of ribbon precursors differs along the pillar/modiolar IHC axis, with a more distinct difference at the IHC extremities. The modiolar IHC fraction contains ribbon precursors that are of lower volume and more plastic than the pillar precursors, and cluster at a closer distance in the cytosol. Also, the modiolar population contains a higher percentage of synaptic precursors and tracks. However, there is no pillar/modiolar difference in precursor number, nor velocity of dynamics. **(B)** Ribbon synapse assembly in IHCs involves three main processes: 1. The engagement of ribbon precursors with the active zone (AZ), through the immobilization and capture of precursors at the AZ, as well as the anchoring to the AZ plasma membrane; 2. The regulation of ribbon precursor volume through events of precursor fusion and fission, in the cytosol (1), as well as at the AZ (2). During these structural plasticity events, precursor aggregates of all detected volumes consist of RIBEYE, Piccolino and Bassoon - hence implying the structural plasticity of complete organelle building blocks; 3. The exchange of ribbon precursor material between different presynaptic AZs, to either reduce or supplement the synaptic contingent, or initiate a novel ribbon precursor/AZ association. **(C)** Presynaptic Ca^2+^ influx appears to be a crucial factor in the developmental plasticity of IHC ribbon precursors, since the blocking of presynaptic Ca_V_s significantly reduces ribbon precursor plasticity at and in proximity of the presynaptic AZ.

In the present study, we established an *in situ* multi-color live-cell imaging approach to monitor the plastic processes taking place during early postnatal development of the mammalian IHC ribbon synapse. We found that ribbon precursors display a high degree of location-dependent spatial mobility and bi-directional structural plasticity (Figure 8). In fact, ribbon precursors translocate in complex interactive networks centered around the presynapse, which captures, confines and exchanges precursors between adjacent AZs–thereby enabling rapid AZ formation at novel sites or structural supplementation of established synapses. We further explored the effects of pre-sensory SA on ribbon dynamics and identified activity-dependent Ca^2+^ influx as a key driver of structural plasticity at auditory ribbon synapses.

### 4.1 Developing ribbon-type AZs are structural plasticity hubs

Based on previous work using “static” imaging methods (Sobkowicz et al., 1982; Sobkowicz et al., 1986; Wong et al., 2014; Michanski et al., 2019) auditory ribbon synapse assembly has been suspected to be a uni-directional process, in which small ribbon precursor spheres self-assemble in the cytosol, gradually accumulate near the AZ to aggregate into larger complexes and eventually form mature, membrane-anchored ribbons (Sobkowicz et al., 1982; Sobkowicz et al., 1986; Hermes et al., 1993; Regus-Leidig et al., 2009; Michanski et al., 2019). Our live-cell study now challenges this simplistic view by revealing the balanced occurrence of precursor fusion and fission events that regulate individual ribbon size in a fluid manner. Importantly, structural plasticity required an intact synaptic context for maximum efficiency. Here, the AZ appears to fulfill two main functions: (i) precursor recruitment and (ii) stabilization for volume regulation. Upon engagement with the AZ, cytosolic precursors are spatially confined, adequately spaced and structurally adapted. Small precursors are regularly exchanged among membrane-resident ribbons and/or precursors that are not (yet) synaptically-engaged, thus leading to dynamic fluctuations in precursor volume, and contributing to the gradual refinement of ribbon shape towards hearing onset (Sobkowicz et al., 1986; Michanski et al., 2019). In concert with such bulk volume adaptations, our findings indicate diffusional addition of soluble RIBEYE to already existing scaffolds, since structurally stable ribbons gradually acquire volume over time. To date, it remains unclear whether this represents separate regulatory pathways or rather reflects fusions and fissions taking place on a scale beyond the resolution limit of our acquisition system. Future use of photo-convertible fluorophores may help clarifying this issue.

### 4.2 Ribbon precursor recruitment to the AZ

During AZ assembly, ribbon precursors undergo various types of mobility that—at first sight—appeared to follow a diffusion-like pattern reminiscent of Brownian motion. However, during approach of the AZ and the exchange of precursors between AZs, small stretches of precursor translocation were characterized by displacement and velocity peaks suggestive of targeted motion. Although not following a rapid, linear trajectory as has been reported for AZ-directed transport at conventional synapses (Hirokawa et al., 2010), it is likely that ribbon precursor dynamics—at least in part—are subject to molecular motor-based transport. Firstly, the variety of ribbon precursor motion types aligns well with the multi-directional microtubule (MT) meshwork of IHCs (Voorn et al., 2024) that contrasts the long-distance presynaptic protein displacement in axonal synapse assembly. Moreover, considering the proximity of MT bundle endings to membrane-attached ribbons, their presumed +end polarity towards the AZ, the colocalization of Kif1a to cytosolic ribbons and reduced ribbon volume accumulation upon Kif1a functional impairment (Michanski et al., 2019; Voorn et al., 2024; Hussain et al., 2025), the IHC MT network may provide one of the key transport routes for ribbon precursor translocation towards the AZ. In fact, MT-based trafficking facilitates precursor fusion and fission, as destabilization of the IHC MTs mainly affected the frequency of plasticity events (Voorn et al., 2024). However, since pharmacological disruption of MTs did not completely halt precursor displacement, additional contributions of other molecular transport pathways, such as the actin cytoskeleton, and/or intracellular processes such as liquid-liquid phase separation (LLPS, see below) can be expected. In the context of IHC presynaptogenesis these present interesting targets for future investigations.

### 4.3 Ribbon precursor confinement at the AZ

Compatible with previous work on retinal photoreceptors (Okawa et al., 2014), our data suggests that the CAZ serves to constrain precursor mobility. Here, the dense precursor clustering ensures sufficient spatial proximity for CAZ/ribbon interactions to take place and—at least transiently—limit ribbon precursor displacement. Such associations likely involve the common suspects, including Bassoon, Ca_V_1.3, RIM2, RIMBP2, and Piccolino (reviewed in Voorn and Vogl, 2020). Interestingly, we found Piccolino and Bassoon to associate with all AZ-proximal precursors, regardless of their specific engagement status with the AZ. Hence, our findings suggest that IHC ribbon precursors serve as “AZ building blocks” prior to AZ attachment, or during intersynaptic exchange. As an essential part of such building blocks, the presence of Bassoon suggests that floating precursors can attach at the plasma membrane easily and thus “seed” ribbons at nascent AZs. However, considering the herein observed transient nature of ribbon precursor contacts with the IHC plasma membrane, sustainable anchoring may require additional post-translational modifications or other currently unknown CAZ interactions. Alternatively, we can speculate that trans-synaptic pairing of cell adhesion molecules may establish focal assembly points for adequate synapse formation, especially since recent findings indicate a role for Neurexin-Neuroligin coupling in ribbon synapse maturation (Ramirez et al., 2022; Jukic et al., 2024). Future studies should investigate this in detail. In this context, it is worth noting that we made multiple observations of AZ-engaged precursor mobility being mirrored by the associated PSD (Supplementary Figure S7), although such instances were too infrequent for meaningful quantification. Nevertheless, such paired ribbon precursor/PSD95 cluster mobility are indicative of the involvement of trans-synaptic coupling to the afferent SGN during IHC synaptogenesis.

### 4.4 Ribbon precursor dynamics differ along the pillar/modiolar IHC axis

The subcellular location of a given ribbon synapse along the pillar/modiolar IHC axis dictates its structural and functional properties (Moser et al., 2023). However, the developmental mechanisms establishing these gradients remain largely elusive. To address this, we examined location-dependent ribbon precursor dynamics in respect to this morphological axis. Interestingly, at the analyzed timepoint at the end of the first postnatal week, modiolar precursors displayed more “synaptic” characteristics, with denser clustering, smaller individual volume, and higher frequency of plasticity events. Accordingly, a larger percentage of modiolar precursors and their associated tracks interacted with nascent AZs than in pillar locations. These findings are hence compatible with reports of mature IHCs presenting with more numerous modiolar synapses, as well as the retention of modiolar multi-ribbon AZs into adulthood (Liberman et al., 2011; Ohn et al., 2016; Michanski et al., 2019; Hua et al., 2021). On the other hand, we detected smaller individual volumes of modiolar ribbon precursors, whereas previous studies found an inverse relationship between pillar and modiolar ribbons in neonatal mice that, in turn, appears to reverse and re-emerge in later stages of development (Liberman and Liberman, 2016; Kalluri and Monges-Hernandez, 2017). Here, our live-cell analysis could add a dynamic new datapoint to the fluctuating timeline described for the pillar-modiolar ribbon volume gradient. Future longitudinal studies on ribbon dynamics will have to be devised to resolve this conundrum.

### 4.5 Intersynaptic exchange facilitates the structural adaptation of IHC AZs

In the context of ribbon anchoring, the frequency of precursor dissociation from the AZ is surprisingly high and often coincided with a peak in precursor velocity. This suggests that a significant energy barrier needs to be overcome to detach precursors from its AZ-anchor or parent ribbon. Here, active processes are likely required to overcome such energy thresholds, as has been shown for the fission of other cellular organelles (Gautreau et al., 2014; Green et al., 2022). Future studies will be required to identify the involved molecular cues and motors.

Such detaching precursors integrated into the highly plastic precursor “crowd” around the resident AZ or supplemented nearby secondary AZs. The latter predominantly constituted directly neighboring synapses, but also included distal synapses that were spatially separated by significant distances (∼7 µm). Even in such cases, the transfer appeared highly directional and targeted. Importantly, intersynaptically exchanged precursors contained the CAZ proteins Piccolino and Bassoon in addition to RIBEYE, and thus can be expected to rapidly modulate the available SV contingent. While exchange of presynaptic proteins and SVs between neighboring boutons has previously been described in neurons—where it gives rise to continuous fluctuations in individual synapse composition (Kalla et al., 2006; Staras, 2007; Tsuriel et al., 2009; Cijsouw et al., 2014)—such re-distribution events occurred with much slower pace than the ones observed here (e.g., >8 h for Bassoon (Tsuriel et al., 2009)). Hence, the assembly into precursor organelles may present a unique and highly efficient mechanism for rapid structural adaptation.

### 4.6 Putative mechanisms of ribbon scaffold assembly

The finding that even the smallest precursors appeared to constitute multi-protein organelles questions how they initially assemble and structurally reorganize during plasticity events. While it is well established that RIBEYE auto-aggregates in the cytosol of sensory receptor cells (Sobkowicz et al., 1986; Hermes et al., 1992; Regus-Leidig et al., 2009; Michanski et al., 2019), it has remained unclear how the other CAZ proteins are recruited to nascent ribbons. RIBEYE aggregates also form in heterologous expression systems (Magupalli et al., 2008), and a recent study could show that—once supplemented with other CAZ components including Bassoon, RIMBP2 and CaV1.3—artificial membrane-anchored ribbons could be generated autonomously (Kapoor et al., 2026). Here, the self-organizing properties of IHC CAZ proteins are reminiscent of nanoclusters/nanocolumn formation at central synapses, where the association of different proteins in biomolecular condensates has been suggested to play a significant role in AZ assembly via liquid-liquid phase separation (LLPS (Choi et al., 2024)). Remarkably, ribbon precursors meet multiple criteria regarding LLPS: (i) precursors are highly dynamic and exhibit a tendency for self-association and spontaneous reversible dispersion, (ii) they occur in mobile and immobile fractions that can exchange material, (iii) ribbons display scaffold-intrinsic mobility, (iv) RIBEYE contains disordered protein sequences and Proline-rich regions, that likely contribute to (v) the ability of RIBEYE to interact with other RIBEYE molecules through various binding sites (Magupalli et al., 2008; Graydon et al., 2017; Chen et al., 2018; Wang et al., 2021). A recent study thoroughly explored the LLPS potential of RIBEYE in heterologous cells and photoceptors, and underlines the likely involvement of phase separation in ribbon plasticity (Liu et al., 2026). Here, especially the intrinsically disordered regions of RIBEYE appear important for the separation into multiple condensates, whereas interactions with synaptic proteins Piccolino and other CAZ components (e.g., CtBP1) are proposed as bidirectional modulators and promote larger cluster stabilization and condensate fragmentation, respectively. Considering the presence of Piccolino at ribbon precursors, as well as the synaptic localization of CtBP1 in auditory IHCs as well as zebrafish lateral line neuromast hair cells (Sheets et al., 2014; Becker et al., 2018), it is tempting to speculate on how local protein levels could affect the bidirectional precursor plasticity and cap ribbon size through phase separation at IHC AZs. Moreover, the plausible role for LLPS in ribbon precursor dynamics allows for a comparison to other biomolecular condensates such as ribonucleoprotein granules, which, analogous to ribbon precursor translocation in mouse and zebrafish hair cells, have been shown to be transported along MTs (Knowles et al., 1996; Voorn et al., 2024; Hussain et al., 2025). The mixed mode of intracellular displacement that would arise from combining molecular-motor based transport and LLPS might explain the highly variable mobility patterns we detected for ribbon precursors during development. Finally, an interesting feature proposed for LLPS taking place at conventional AZs is the implication of two functional pathways: one for the clustering of Ca_v_s and one for SV organization (Emperador-Melero et al., 2024). The unique position of the synaptic ribbon might bridge these functionalities by clustering Ca_v_1.3 and facilitating SV priming and replenishment (LoGiudice et al., 2008; Sheets et al., 2011; Snellman et al., 2011). Altogether, this makes for an enticing argument for ribbon precursors as biomolecular condensates, and the contribution of LLPS to IHC synapse assembly.

### 4.7 Activity-dependent plasticity of developing ribbon synapses

Finally, we investigated a functional link between ribbon plasticity and spontaneously-occurring pre-sensory SA. Considering the strong interconnectivity between anchored ribbons, CAZ proteins and Ca_v_ clusters, it is conceivable that activity-dependent Ca^2+^ influx and/or the associated membrane turnover could alter ribbon dynamics. In fact, previous studies reported a correlation between synaptic activity and Ca_v_ cluster mobility along the presynaptic membrane of retinal photoreceptors (Mercer et al., 2011). We found that pharmacological silencing of spontaneous presynaptic Ca^2+^ influx significantly reduced precursor velocity and attenuated the structural plasticity at the AZ, as synapse-interacting tracks displayed plasticity profiles comparable to non-synaptic precursors, thus implicating Ca_v_1.3-mediated Ca^2+^ influx as a major driver of this process. Interestingly, various molecular motors have been shown to be regulated by local Ca^2+^ levels, to either associate or disassociate from the cargo or the cytoskeleton (Hirokawa et al., 2010). Likewise, the formation of distinct liquid states during LLPS can depend on local increases in Ca^2+^ levels (Zhu et al., 2024), thus rendering both mechanisms as plausible candidates for the observed bi-directional plasticity of ribbon scaffolds.

Notably, at conventional synapses, synaptic activity has been shown to exert differential effects on the presynaptic protein complement: while Munc-18 and Synapsin were redistributed between synapses in an activity-dependent manner (Chi et al., 2001; Cijsouw et al., 2014), Munc-13 and Bassoon remained unaffected. This indicates a mechanistic distinction between soluble SV-associated proteins and membrane-anchored scaffolds and places the ribbon in a unique position, being involved in both pathways. However, pharmacological presynaptic silencing did not only affect ribbon precursors in close proximity to the AZ, but indiscriminately reduced precursor velocity. The disruption of ribbon precursor dynamics regardless of their synaptic engagement suggests that this effect is separate from the AZ-centered attenuation of structural plasticity, and likely involves a fundamental molecular pathway of IHC homeostasis.

Despite the differential ribbon dynamics, the loss of presynaptic Ca^2+^ influx minimally affected ribbon precursor volume and NND. This is an interesting comparison to findings in zebrafish that demonstrate ribbon volume to increase upon the genetic or pharmacological inhibition of presynaptic Ca^2+^ influx (Sheets et al., 2012; Wong et al., 2019). However, the effect was considerably stronger in young, compared to more mature nHCs, which suggests that the developmental time window for the impact of SA loss may appear relatively early and cease with advancing age. This scenario is therefore compatible with the observed relative rigidity of ribbon structure in response to pharmacological inhibition of Ca^2+^ influx herein and in our previous work (Halim et al., 2026). The latter moreover indicated that the homeostatic scaling of presynaptic ribbons may be more sensitive to increased IHC activation rather than inhibition, and that the temporal patterning of the SA is crucial. Hence, the combination of optogenetic stimulation of IHCs with live-cell imaging of ribbon dynamics is an exciting prospect, and will be the focus for future experiments.

Finally, the apparent correlation between presynaptic Ca^2+^ levels and precursor plasticity is of interest in the context of the location-dependent plasticity we detected along the pillar/modiolar IHC axis: The highly plastic and more “synaptic-like” characteristics of ribbon precursors in the modiolar IHC fraction imply a potentially higher level of Ca^2+^ influx driving this difference. This would align with the higher number and volume of modiolar ribbon synapses found in mature IHCs, which have been shown to correlate with the amplitude of Ca^2+^ influx, number of Ca_v_s, and their activation thresholds (Frank et al., 2009; Liberman and Liberman, 2016; Ohn et al., 2016; Neef et al., 2018; Hua et al., 2021; Özçete and Moser, 2021). However, it remains unclear whether any heterogeneous Ca_v_ expression pattern is already present during early development and if distinct innervation may retrogradely initiate the differentiation of modiolar AZs. Yet, the exact driver of such reciprocal pre- and postsynaptic influence remains to be determined.

Together, our data substantiate previous *in vivo* work indicating intrinsic synaptic differences along the pillar/modiolar axis during early development (De Faveri et al., 2025) and this pre-determined polarity appears fundamental for adequate AZ development.

### 4.8 Technical limitations of this study

The cochlea is tightly embedded in the temporal bone and hence inaccessible for high-resolution long-term optical analysis of individual ribbon synapse complexes *in vivo*. Thus, we opted for a short-term organotypic culture approach to study the mechanisms of synaptogenesis in an accessible and tightly controlled semi-intact system. While our organotypic cultures retained excellent structural integrity and afferent innervation, the dissection and culturing procedure inevitably causes amplified SGN loss compared to the described pruning that occurs naturally *in vivo* (Huang et al., 2007; Barclay et al., 2011; Halim et al., 2026). However, through limiting our analyses to a brief and clearly defined time window subsequent to tissue extraction and carefully monitoring cell health and global structural morphology during image acquisition, the dissection trauma could be mitigated as indicated by the overall well-preserved structural organization, stable neuronal survival and even targeted outgrowth of type II SGNs (Supplementary Video S1) and retained intrinsically-generated spontaneous activity waves (Supplementary Video S13). While these precautions enabled us to conduct detailed analyses of developing IHCs *in situ*, we faced several other methodological limitations that need to be considered when interpreting our data: Firstly, to avoid activating the ChR2 expressed in the IHC membrane we employed a 515 nm laser to excite the linked -YFP for cellular context visualization as this wavelength resides at the very end of the ChR2 activation spectrum (optimum ∼470 nm). Considering the high power densities required to evoke IHC photo-depolarizations (Chakrabarti et al., 2022; Halim et al., 2026), and the comparable ribbon dynamics profiles we detected between the different mouse-lines (Ai14-Neurog1-PSD and Ai3-VC-KI-PSD), live-cell imaging mediated optogenetic IHC photoactivation is likely to be negligible. Secondly, the temporal resolution of multi-color 3D data acquisition is severely restricted by the sequential fluorescence channel acquisition. Therefore, ultrafast events will have failed to register in our experiments and rapid displacements or transient plasticity events may have been missed. Future studies should hence opt for high-speed imaging techniques to extend our knowledge on synaptic protein dynamics during IHC synaptogenesis. Thirdly, the Halo-based PSD labeling was variable between experiments and differed in signal-to-noise ratio (i.e., the JF549 ligand consistently outperformed the JF646 ligand in our triple-color experiments). Hence, we cannot exclude the possibility that dimly fluorescent PSDs went undetected, or that ribbon precursors therefore have been misclassified as “membrane-proximal” instead of “synaptically-engaged” in our live-cell experiments. In combination with the dissection-related SGN loss due to culturing procedures, the synaptic fraction of precursors we observe will likely additionally be reduced in comparison to IHCs of this developmental age *in vivo*. Although this may minimally alter the population statistics of membrane-proximal precursors, it would not affect the synaptic ribbon precursor class that partner a bright, distinguished PSD. Finally, at this point, our data provide a teaser of the dynamic processes underlying IHC presynaptogenesis by focusing on a transient period at the end of the first postnatal week. Future studies should seek to perform longitudinal analyses with multiple age groups in the runup to—and surpassing—hearing onset to generate a clear and comprehensive picture of IHC synapse development.

## Data Availability

The datasets presented in this study can be found in online repositories. The names of the repository/repositories and accession number(s) can be found in Table 1.
