# Supplementary material for: Dynamic structural plasticity facilitates the developmental assembly of cochlear inner hair cell ribbon synapses
Source: Front Cell Dev Biol. 2026 Sep 4;14:1866692. doi: 10.3389/fcell.2026.1866692 (PMC13585721; doi:10.3389/fcell.2026.1866692)
Supplement: Supplementary file 1 [file DataSheet1.pdf]

Dynamic structural plasticity facilitates the developmental assembly of cochlear inner hair cell ribbon synapses

Authors: Roos A. Voorn[1,2,3,4], Michael Sternbach[4,5,6], Jérôme Bourien[7], Noboru H. Komiyama[8,9,10,11], Vladan Rankovič[12#], Fred Wolf [3,5,6,13], Seth G. N. Grant[8,9], Christian Vogl [1,2,3,*]

Supplementary Material

This PDF file includes:

Supplementary Methods

Figures S1-S7

Table S1

Videos S1-S13

1 Supplementary Methods

Supplementary Methods for Figures S2, S3, S5, S6 and Table S1.

Figure S2.

Organotypic cultures of the organ of Corti of mice on postnatal day (P)3, P5 and P7 and were kept for 1 day in vitro (DIV). Acute preparations of the organ of Corti were also performed on DIV3, DIV5 and DIV7. Both types of preparations were fixed on ice in 4% formaldehyde (in PBS) for 15 minutes, and thereafter processed for immunohistochemical stainings for Myosin VIIa, Ribeye-A, and PSD95. Immunostained samples were imaged using an Abberior Instruments Expert Line STED microscope – operated in confocal laser scanning mode, using the 100x/NA 1.4 oil immersion objective, with a pixel size of 80 nm xy and 150 nm in z. The images were processed in IMARIS using the IMARIS surface rendering function, for ribbon precursors (0.054 surface detail, automatic local background subtraction, 0.28 µm largest sphere diameter, region growing diameter 0.15 µm, 3 quality filter) as well as postsynaptic densities (0.08 surface detail, automatic local background subtraction, 0.6 µm largest sphere diameter, 3 quality filter). Ribbon precursors were classified as “synaptic” when localized in proximity of 500 nm or less to the rendered PSD, and as cytosolic when at a larger distance. These values were then exported and analyzed in Prism8 and 10 (GraphPad, San Diego, CA).

Figure S3 and S6.

<u>Statistics</u>

For the normally distributed cell average values in Supp. Fig.S3C-E, values are compared with a repeated-measures ANOVA with Tukey’s multiple comparisons test.

For the cell average values in Supp. Fig.S6, the fractional change per IHC for each condition post-treatment is calculated, based on the difference in cell average pre- and post-treatment. Due to an unequal amount of cells per condition, a Kruskal-Wallis test was used to compare the values.

Figure S5.

<u>Viral transduction</u>

Viral transduction *in vitro* was performed on P3/DIV0 or P4/DIV0 cultures of SNAP25-GCaMP6 mice, by application of PHP.B_HBA_Katushka-RIBEYE-BP_WPRE_bGHpA (1.5e+11 gc/mL) and incubation at 37C 5% CO_2 for ≥72 hours (DIV3) to allow for sufficient time for fluorophore expression.

<u>Short-term high-speed dual-color time-lapse imaging</u>

Short-term time-lapse imaging experiments were performed at an Abberior Instruments Expert Line STED microscope, operated in confocal laser scanning mode, using a 60x/1.20 NA water immersion objective. A top mount on-stage incubator (Okolab uno stage top incubator, H391-Olympus-IX-SUSP 2015) was used to create and maintain environmentally-controlled conditions (37°C, 5% CO_2). ROI selection was based on GCaMP6s signal strength and fluorescence periodicity, plus detection of the Katushka-RIBEYE-BP expression levels to be sufficient for imaging. Dual-color time-lapse images

were acquired over a 5 or 10-minute period, and consisted of single-plane optical sections, at intervals of 0.75 seconds. Spatial dimensions of the regions for either imaging condition were minimized and adapted to tissue orientation, at approximately 15x15 µm. The two colors were acquired in confocal mode, by sequential line scanning, 2 times accumulation and a 5 ns dwell time.

Time-lapse image processing and analysis

The fluorescent GCaMP6s signal was processed in FIJI/ImageJ, by extraction of the fluorescence intensity (F) over time. To create the ROI, an image mask was generated based on the maximum intensity projection, followed by intensity thresholding and noise reduction. The extracted fluorescence intensity was then baseline corrected ($\Delta F = F - F_0$). Ribbon precursor velocity was traced in IMARIS, using the Spots and Lineage tracing function. Potential synchronicity between peaks in the GCaMP6s fluorescence and ribbon precursor velocity was analyzed using custom MATLAB scripts.

Table S1.

The correlation between RIBEYE-tdTomato expression levels and extracted ribbon precursor parameters in Supplementary Table S1 was performed based on the average values per IHC. The RIBEYE-tdTomato expression level per IHC was determined by summation of all signal over time for a predetermined ROI and averaging over the ROI volume. Values were correlated with a Pearson's R correlation matrix.

2 Supplementary Figures and Tables

2.1 Supplementary Figures

Fig. S1.

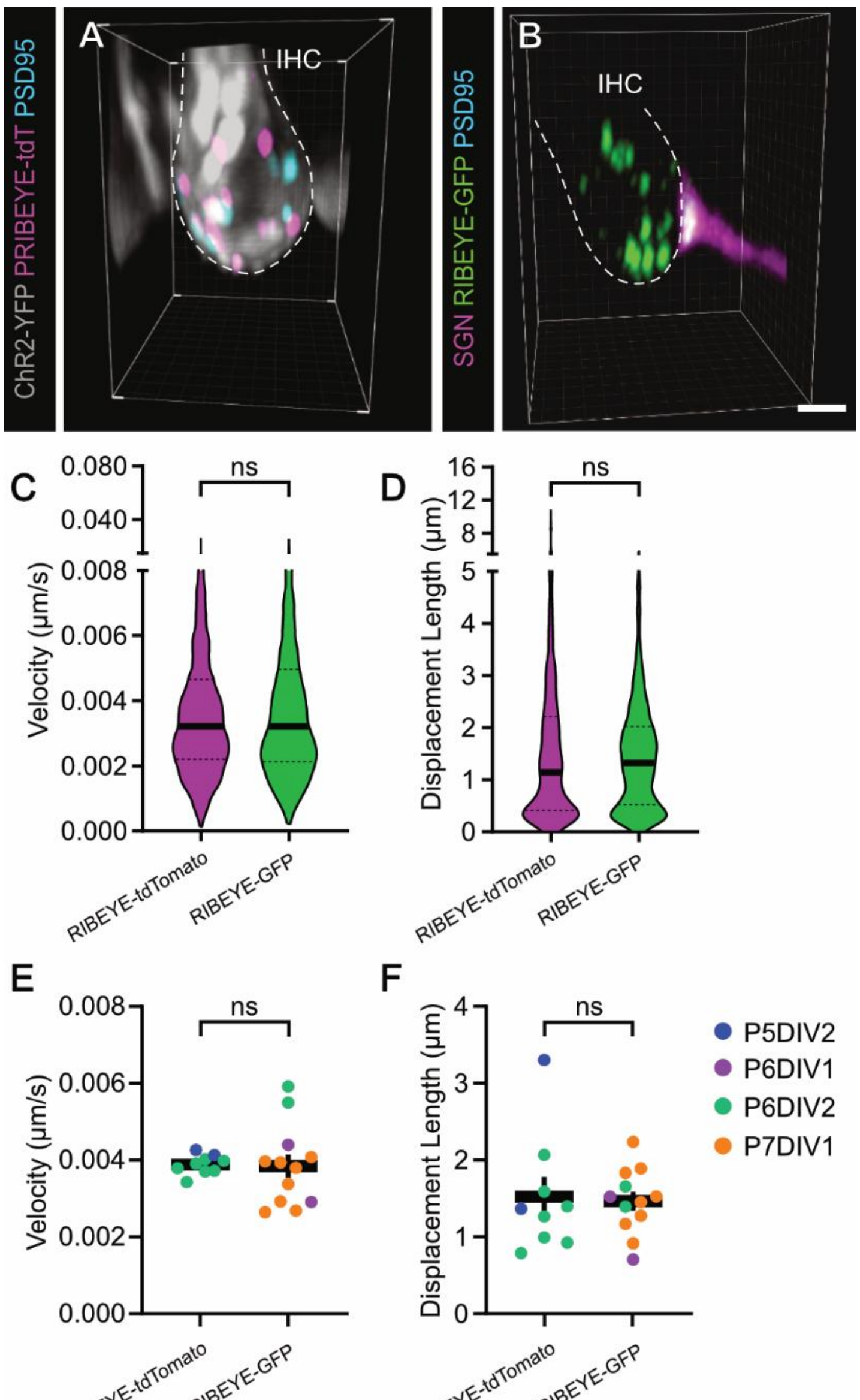

Figure S1. Overexpression of RIBEYE-GFP and RIBEYE-tdTomato result in comparable synaptic ribbon precursor dynamics. A. 3D representation of a live-cell imaged organotypic culture of the organ of Corti of an Ai32-VC-KI-PSD mouse (P6DIV2), with overexpressed RIBEYE-tdTomato and labeling of the PSD through JF646. **B.** 3D representation of a live-cell imaged organotypic culture of the organ of Corti of an Ai14-Neurog1-PSD mouse (P6DIV2), with overexpressed RIBEYE-GFP and labeling of the PSD through JF646. The (presumed) IHC membrane is indicated by a white dashed line. **C, D.** The velocity (**C**) as well as the displacement length (**D**) of synaptic ribbon precursors is comparable between the two RIBEYE-xFP overexpression conditions. **E, F**. When comparing the average values per IHC for the ribbon precursor velocity (**E**) and displacement (**F**) there is no difference

between the two experimental setups. The different mouse ages and culturing times are indicated. We can observe a considerable variability between the individual IHCs, with a notably limited spread in synaptic ribbon precursor velocity in the experimental setup employing RIBEYE-tdTomato. Statistical significance: Mann-Whitney U. $N_{animals}$=11, n_{IHC}=21, $n_{ribbons}$=4678. Scale bar: 5 µm.

Fig. S2.

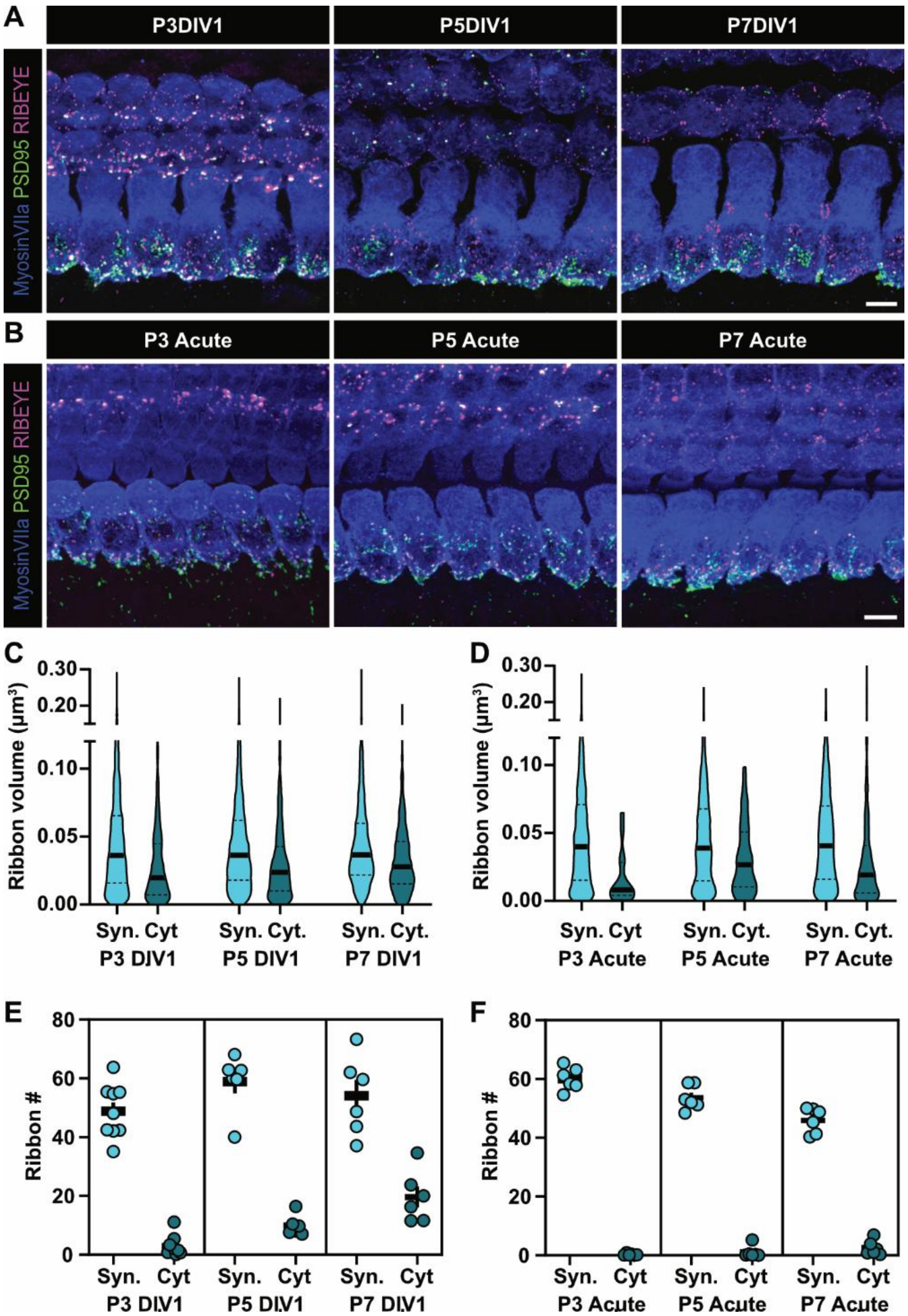

Figure S2. Ribbon precursor number and volume in organotypic cultures and acutely dissected organs of Corti. A-B. Exemplary confocal images used for the analyses of ribbon precursor volumes and numbers in DIV1 organotypic cultures (**A**) and acutely dissected (**B**) organs of Corti of different developmental ages. **C-D**. The volume of ribbon precursors classified as either synaptic or cytosolic for different ages in organotypic cultures (**C**) and the age-matched acute preparations (**D**). No differences were found when comparing DIV1 and acute preparations, for synaptic nor non-synaptic precursors, for none of the presented ages. **E-F.** The number of cytosolic ribbons increases slightly with developmental age in the cultured preparations (**E**), whereas in acute preparations, almost all ribbon precursors remain synaptically-engaged (**F**). However, this trend was not statistically significant. On the other hand, the number of synaptic ribbon precursors might reduce over time in the

acute preparations, while in the organotypic cultures, there is a minimal change. Again, this trend was not statistically significant. Statistical significance: Kruskal-Wallis test. $N_{animals}$=12, n_{Corti}=20, n_{IHC}=38, $n_{ribbons}$= 20218. Scale bars: A, B: 5 µm.

Fig. S3.

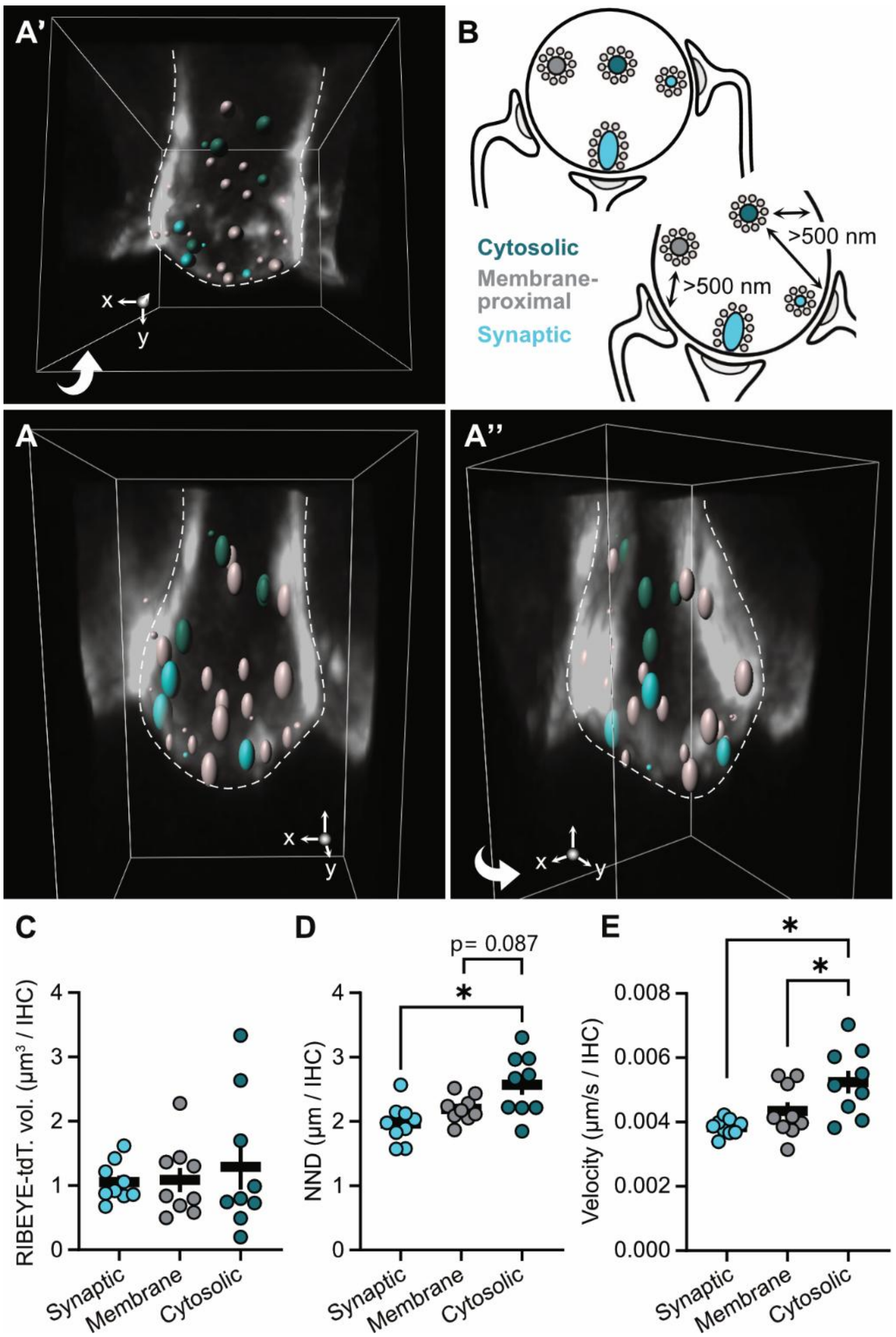

Figure S3. Ribbon precursor classification and determination of intracellular localization within the 3-dimensional IHC. (A) Representative image of a 3D multi-color imaged IHC, with indicated ribbon precursor localization within the IHC. The different colored spheres represent the different identified precursor classes**. (A')** top down and **(A'')** turned side view of the IHC in A to illustrate the localization of the different precursor classes in respect to the IHC plasma membrane. (**B)** Graphical illustration defining the three precursor classes based on a 500 nm distance cut-off from either the synaptic contact or IHC membrane, for the side view as well as the top-down view. (**C)** When

comparing the average values per IHC for the three different precursor classes, ribbon precursor volumes are comparable. (**D)** The average nearest neighbor distance (NND) per IHC is significantly higher for cytosolic precursors compared to synaptic precursors, demonstrating the trend for precursor crowding within the presynaptic active zone (AZ) context. Also, we detect a trend towards a statistically significant difference between membrane proximal and cytosolic precursors. (**E**) The average velocity of synaptic precursors per IHC is significantly lower than of membrane-proximal and cytosolic precursors, indicating a reduction in precursor mobility at the AZ. *$p<0.05$. Statistical significance: Repeated-measures ANOVA. $N_{animals}=4$, $n_{IHC}=9$.

Fig. S4.

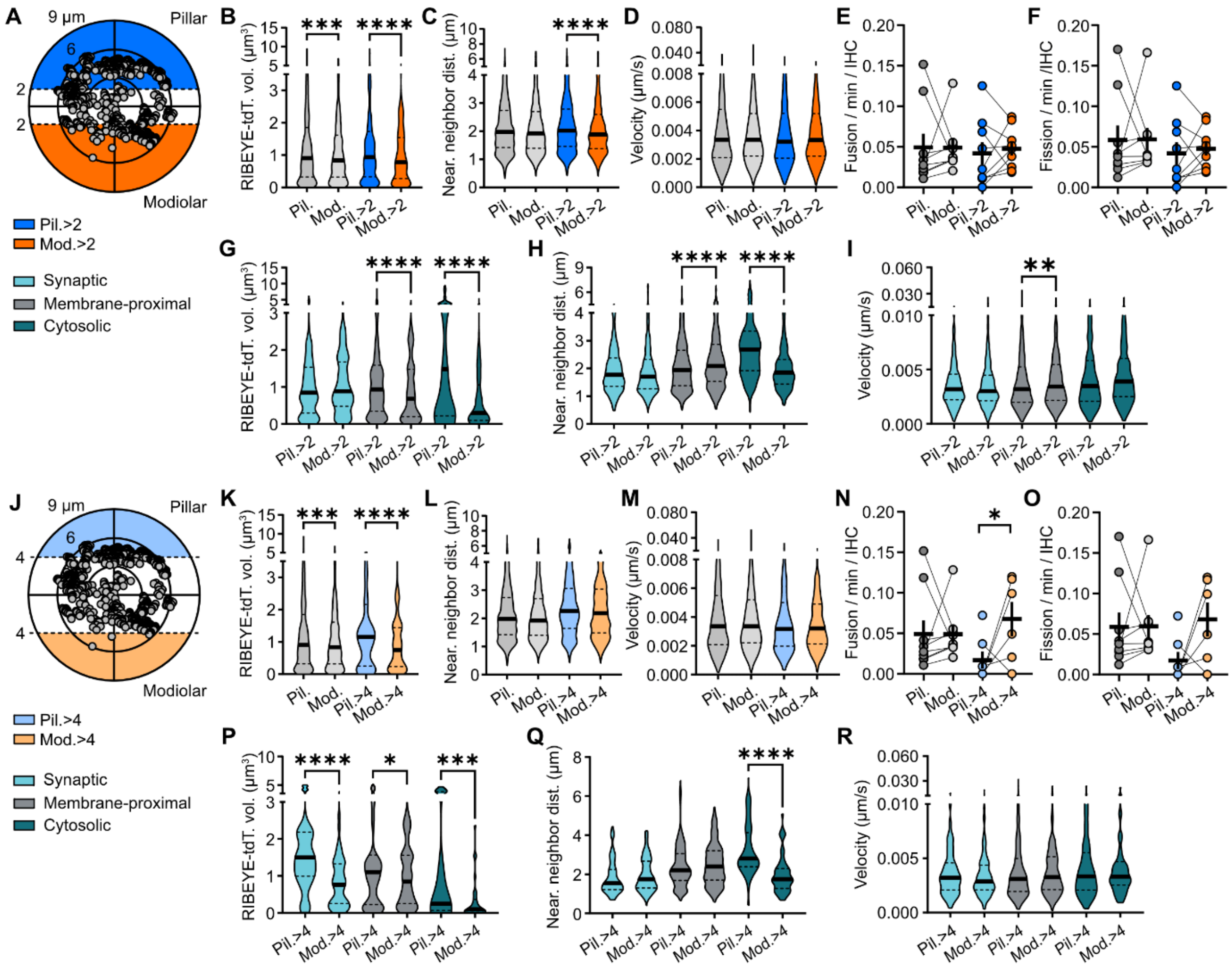

Figure S4. Ribbon plasticity along the pillar/modiolar IHC axis, cutoffs 2 µm and 4 µm. A. Illustration of the >2 µm extremities within the axes of the IHC; all precursors over time are displayed. **B-F.** Grays: pillar/modiolar division based on the 0 midline, blue/orange: pillar/modiolar populations taken from the >2 µm extremities. **B.** The volume of modiolar ribbon precursors is lower than of pillar precursors. **C.** The nearest neighbor distance (NND) of modiolar precursors is lower than of pillar precursors in the >2 µm extremities. **D.** Ribbon precursor velocity is comparable throughout the IHC. **E, F.** The frequency of fusion (**E**) and fission (**F**) events per minute per IHC is comparable in modiolar and pillar tracks in the >2 µm IHC extremities. **G-I.** Specifically for the >2 µm extremities, considering the three different precursor classes: synaptic (cyan), membrane-proximal (gray), and cytosolic (teal) precursors. **G.** The volume of membrane-proximal and cytosolic modiolar precursors was lower than of pillar precursors. **H.** The lower NND of the >2 µm modiolar precursor population appears to be driven by the cytosolic precursors, whereas the membrane-proximal precursors are in fact at a slightly higher NND. **I.** The ribbon precursor velocity was slightly higher for modiolar membrane-proximal precursors than pillar, and showed a similar trend for the cytosolic precursor population ($p=0.0518$), in the >2 µm pillar/modiolar IHC extremities. **J.** Illustration of the >4 µm extremities within the axes of the IHC; all precursors over time are displayed. **K-O.** Grays: pillar/modiolar division based on the 0 midline, light blue/orange: pillar/modiolar populations taken from the >4 µm extremities. **K.** The volume of modiolar ribbon precursors is lower than of pillar precursors. **L.** The NND of modiolar precursors does not differ from pillar precursors in the >4 µm extremities. **M.** Ribbon precursor velocity

is comparable throughout the IHC. **N.** The frequency of fusion events is higher in the modiolar >4 µm IHC extremities than the pillar. **O.** The number of fission events per minute per IHC shows a trend towards a higher frequency in the modiolar than pillar tracks in the >4 µm IHC extremities ($p=0.0641$). **P-R.** Specifically for the >4 µm extremities, considering the three different precursor classes. **P.** The volume of modiolar precursors was overall lower than of pillar precursors. **Q.** The NND of the >4 µm modiolar precursor only differs from the pillar population in the cytosolic precursor class. **R.** The ribbon precursor velocity was similar for all precursor classes in the >4 µm pillar/modiolar IHC extremities. *$p<0.05$, **$p<0.01$, ***$p<0.001$, ****$p<0.0001$. Statistical significance: Kruskal-Wallis. $N_{animals}=4$, $n_{IHC}=9$, $n_{ribbons}=10855$.

Fig. S5.

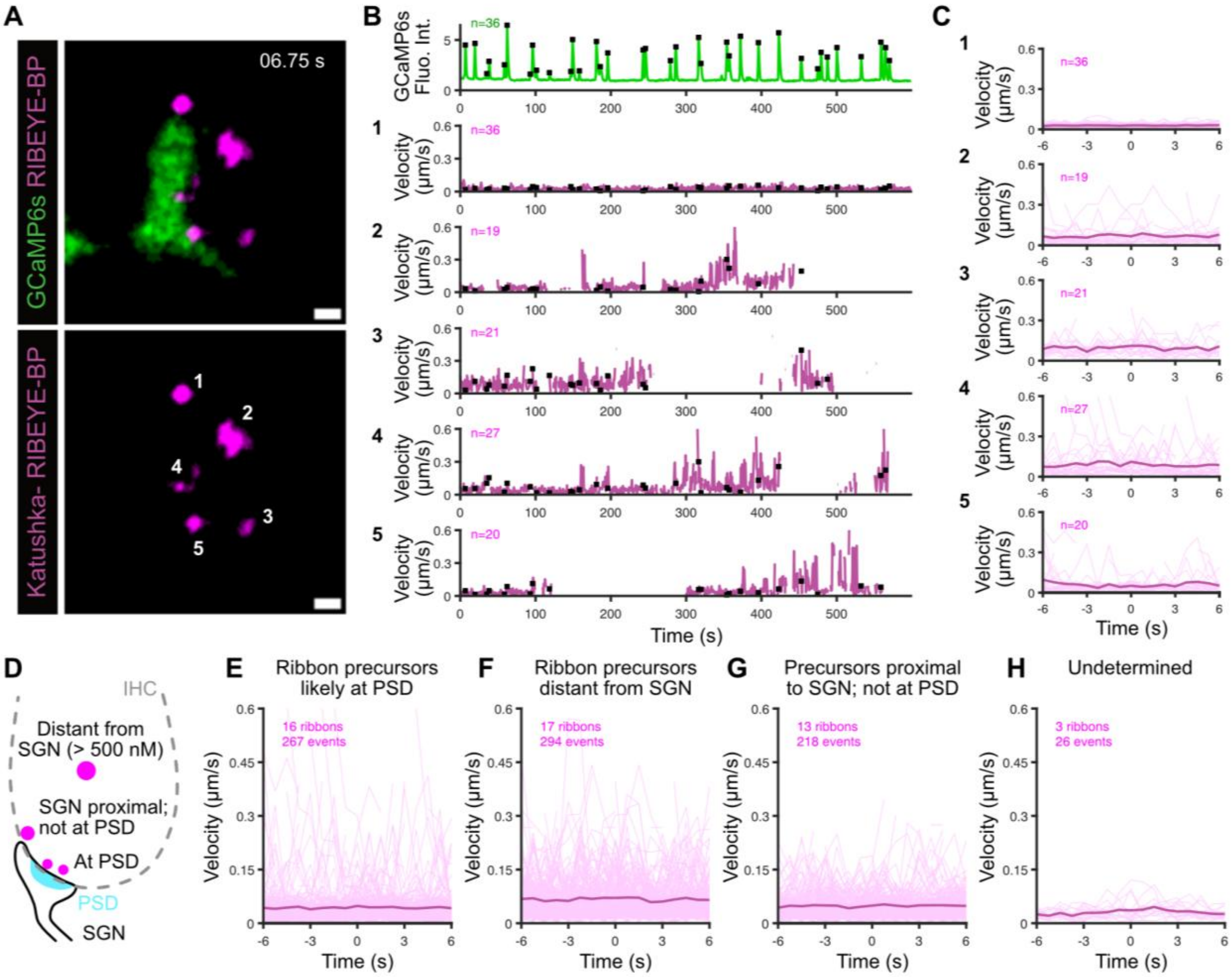

Figure S5. Ribbon precursor dynamics do not correlate with postsynaptic SGN activity. A. Exemplary live imaged frame of an IHC expressing Katushka-RIBEYE-binding peptide (P3DIV3), innervated by a GCaMP6s expressing afferent SGN. The different traced ribbon precursors are indicated by number. **B.** GCaMP6s fluorescence intensity was traced over time as a postsynaptic measure of spontaneous synaptic activity and exocytotic release of the IHC. Simultaneously, the dynamics of the ribbon precursors indicated in A were traced, plotted here is the object velocity over time. Interludes of trace absence likely reflect the displacement in Z of the ribbon precursor, out of the imaged ROI (single plane). **C.** Spike-triggered averaging of velocity traces in B using the GCaMP6s fluorescence as trigger (12 s time window). The number of events used for averaging is indicated by 'n', reflecting the number of fluorescence peaks that could be correlated to ribbon precursor velocity values. **D.** Schematic illustration of different ribbon precursor categories based on their intracellular location. **E-H.** Grand averages of spike-triggered velocity traces for all traced ribbon precursors of the different categories in D: precursors (**E**) localized in probable association with a PSD; (**F**) at a distance (> 500 nM) from the fluorescent afferent SGN; (**G**) proximal to the innervating SGN, but localized where partnering with a PSD is highly unlikely; (**H**) of undetermined location. The absence of a reproducible pattern suggests statistical independence between fluorescence dynamics and ribbon mobility, in all precursor locations. $N_{animals}$= 3, N_{IHC}= 8, $n_{ribbons}$= 49. Scale bars: 1 µm.

Fig. S6

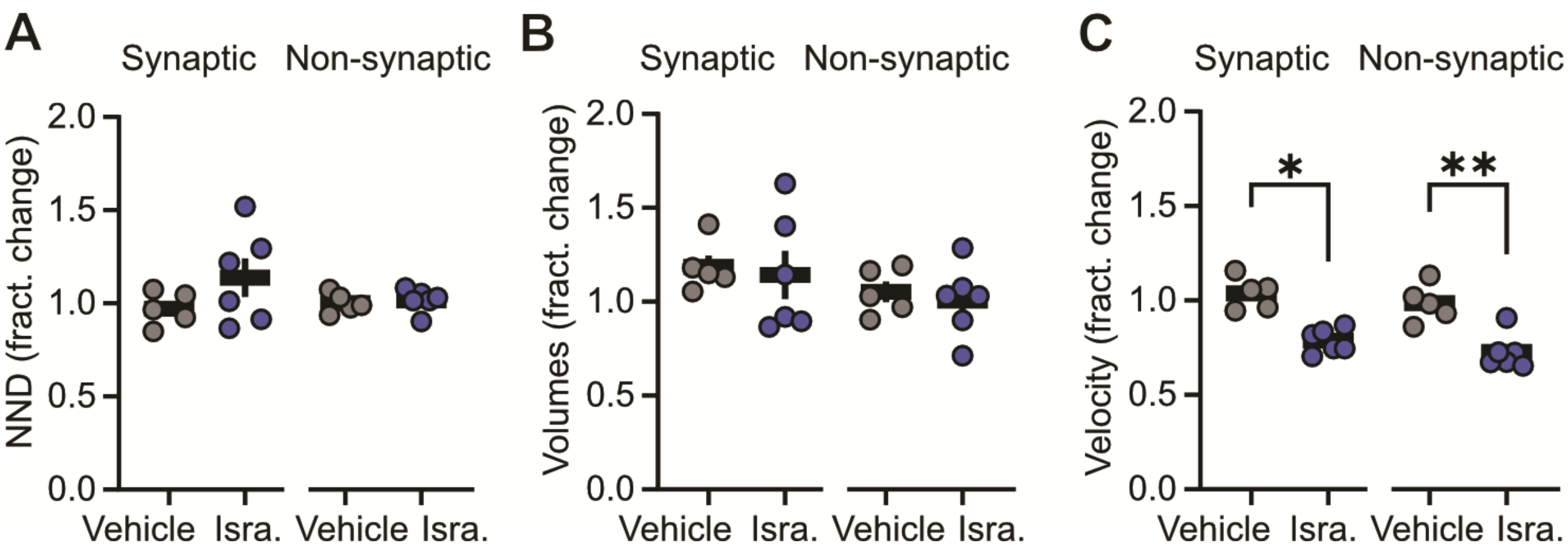

Figure S6. Average fractional change per IHC upon isradipine treatment. A. Assessment of the fractional change in nearest neighbor distance (NND) per IHC for the synaptic and non-synaptic ribbon precursor populations shows no difference between isradipine treatment conditions and the vehicle (DMSO) control. **B.** There is no fractional change in the IHC averages for ribbon precursor volume when comparing isradipine treatment to the vehicle control. **C**. The average values per IHC demonstrate the reduction in ribbon precursor velocity upon isradipine treatment, in the synaptic as well as non-synaptic population. *$p<0.05$, **$p<0.01$. Statistical significance: Kruskal-Wallis. $N_{animals}=7$, $n_{IHC}=11$.

Fig. S7.

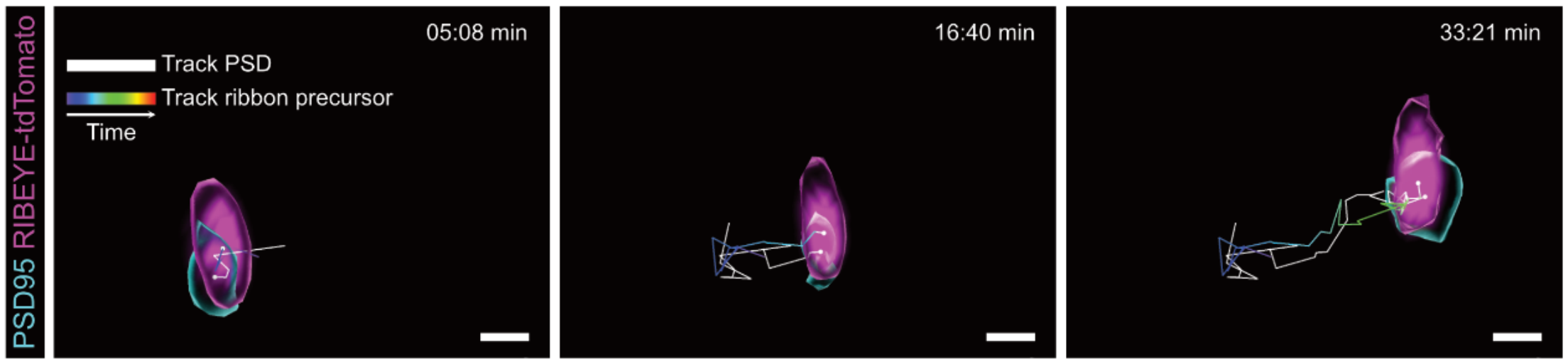

Figure S7. Co-mobility of a transsynaptically-coupled ribbon precursor and PSD. Exemplary 3D live imaging frames of a ribbon precursor (magenta) engaged at the presynaptic AZ and PSD cluster (cyan), traced during coupled displacement. The aligned tracks are shown in white (PSD) and a rainbow color gradient (ribbon precursor), reflecting the time. Scale bar: 1 µm.

Table S1.

Table S1. Correlation of the average RIBEYE-tdTomato expression level per IHC to the different analyzed parameters of ribbon dynamics

	Pearson r	95% conf. interval	R squared	P (two-tailed)	P summary	Significance (alpha = 0.05)	Number of XY Pairs
**Average RIBEYE-tdTomato expression level / IHC correlated to:**							
Fission frequency synaptic tracks	-0,05003	-0,6912 to 0,6352	0,002503	0,8983	ns	No	9
Fission frequency membrane-proximal tracks	-0,4528	-0,8587 to 0,3022	0,205	0,221	ns	No	9
Fission frequency cytosolic tracks	-0,5937	-0,9307 to 0,2883	0,3524	0,1599	ns	No	7
Fusion frequency synaptic tracks	-0,06483	-0,6989 to 0,6263	0,004203	0,8684	ns	No	9
Fusion frequency membrane proximal tracks	-0,4621	-0,8618 to 0,2915	0,2135	0,2105	ns	No	9
Fusion frequency cytosolic tracks	-0,4642	-0,9020 to 0,4441	0,2155	0,2941	ns	No	7
NND synaptic precursors	-0,0773	-0,7052 to 0,6186	0,005975	0,8433	ns	No	9
NND membrane-proximal precursors	0,3132	-0,4431 to 0,8090	0,09807	0,4119	ns	No	9
NND cytosolic precursors	0,2053	-0,5312 to 0,7651	0,04216	0,5961	ns	No	9
Velocity synaptic precursors	0,1832	-0,5475 to 0,7554	0,03356	0,6371	ns	No	9
Velocity membrane-proximal precursors	-0,5426	-0,8871 to 0,1899	0,2945	0,1312	ns	No	9
Velocity cytosolic precursors	-0,3693	-0,8299 to 0,3906	0,1364	0,328	ns	No	9
Volume synaptic precursors	0,7422	0,1539 to 0,9420	0,5508	0,022	*	Yes	9
Volume membrane-proximal precursors	0,9438	0,7493 to 0,9884	0,8907	0,0001	***	Yes	9
Volume cytosolic precursors	0,8539	0,4384 to 0,9687	0,7292	0,0034	**	Yes	9

2.2 Supplementary Videos

Video S1. Long-term time-lapse imaging of tdTomato expressing SGN in organotypic cultures of the organ of Corti

Time-lapse video of an organotypic culture of the organ of Corti of an Ai14-Neurog1-PSD mouse (P5DIV2), imaged for 12 hours, every 15 minutes. The endogenous sparse tdTomato labeling of the SGN allows for the monitoring of individual fibers, for which we can observe the continuous structural plasticity and refinement in the postsynaptic terminals of the type I SGN, as well as the fiber outgrowth in the type II SGN.

Video S2. Ribbon plasticity within the IHC

Time-lapse video of an IHC of the Ai32-VC-KI-PSD mouse line, expressing RIBEYE-tdTomato (magenta), P6DIV2. The IHC membrane is indicated by the ChR2-YFP expression (gray) and the PSD is labeled through HaloTag ligand JaneliaFluor 646 (cyan, JF646). The highly dynamic ribbon precursors are traced over 90 minutes, 77 s / frame; color coded in the rainbow LUT for time.

Video S3. Ribbon plasticity within the IHC without surface rendering

Video S2 without rendering of the PSD surfaces.

Video S4. Example of ribbon precursor fusion

Time-lapse video example of two ribbon precursors (magenta) fusing while located close to the rendered PSD (cyan), thereby rendering it highly likely to occur at the presynaptic AZ.

Video S5. Example of ribbon precursor fusion without surface rendering

Video S4 without rendering of the PSD or ribbon precursor surfaces.

Video S6. Example of a continuously synaptic ribbon precursor

Time-lapse video example of a ribbon precursor (magenta) that continuously localizes in close proximity to the (dynamic) PSD (cyan), thus showing sustained association with the presynaptic AZ.

Video S7. Example of a continuously synaptic ribbon precursor without surface rendering

Video S6 without rendering of the PSD or ribbon precursor surfaces.

Video S8. Example of ribbon precursor fission

Time-lapse video example of the fission of one ribbon precursor into two smaller precursors (magenta) with individual dynamics, located at the AZ.

Video S9. Example of ribbon precursor fission without surface rendering

Video S8 without rendering of the PSD or ribbon precursor surfaces.

Video S10. Intersynaptic exchange facilitates ribbon supplementation

Time-lapse video of an intersynaptic exchange event taking place within the IHC basolateral compartment, in which ribbon material of a primary AZ is translocated to – and ultimately fuses with – a nearby secondary AZ. The track of the exchanged ribbon fragment is temporally-coded for better visualization. PSDs were live-labelled with HaloTag ligand JF549 (cyan), presynaptic ribbons via AAV-mediated expression of RIBEYE-GFP (magenta), in an Ai14-Neurog1-PSD (cre-) P5DIV2 organotypic culture.

Video S11. Intersynaptic exchange between ribbon synapses occurs over large intracellular distances and is strikingly targeted

Time-lapse video of a long-distance intersynaptic exchange event, in which an initially stable synaptic complex disintegrates and the detached ribbon translocates to a secondary AZ at the base of the IHC, its trajectory is illustrated with a temporally color-coded track. Interestingly, the translocated ribbon does not fuse with the resident ribbon, but rather settles in a new spot at the same PSD patch. PSDs were live-labelled with HaloTag ligand JF646 (cyan), presynaptic ribbons via AAV-mediated expression of RIBEYE-tdTomato (magenta), the IHC plasma membrane is outlined by ChR2-YFP expression (gray), in an Ai32-VC-KI-PSD P5DIV2 organotypic culture.

Video S12. Intersynaptic exchange between ribbon synapses occurs over large intracellular distances and is strikingly targeted, without surface rendering

Video S11 without rendering of the PSD surfaces.

Video S13. Spontaneous activity in organotypic cultures of the organ of Corti

Time-lapse movie of the intrinsic spontaneous activity present in the developing organ of Corti of a SNAP25-GCaMP6 mouse that was maintained in organotypic culture (this culture: P5DIV1). Here, the GCaMP6s expression in the SGN enables the readout of postsynaptic activation triggered by the spontaneous release by the IHCs. Video over 5 minutes, imaged every 0.78 s.
